# Impacts of fertilization methods on *Salvia miltiorrhiza* quality and characteristics of the epiphytic microbial community

**DOI:** 10.3389/fpls.2024.1395628

**Published:** 2024-05-16

**Authors:** Feng Gong, Chao He, Xianen Li, Kehan Wang, Min Li, Xiangyun Zhou, Minghui Xu, Xueli He

**Affiliations:** ^1^College of Life Sciences, Hebei University, Baoding, China; ^2^Key Laboratory of Microbial Diversity Research and Application of Hebei Province, Baoding, China; ^3^Institute of Medicinal Plant Development, Chinese Academy of Medical Sciences and Peking Union Medical College, Beijing, China

**Keywords:** core microbiome, plant–microbe interactions, deterministic processes, hormones, community assembly

## Abstract

Plant epiphytic microorganisms have established a unique symbiotic relationship with plants, which has a significant impact on their growth, immune defense, and environmental adaptation. However, the impact of fertilization methods on the epiphytic microbial community and their correlation with the yield and quality of medicinal plant was still unclear. In current study, we conducted a field fertilization experiment and analyzed the composition of epiphytic bacterial and fungal communities employing high throughput sequencing data in different organs (roots, stems, and leaves) of *Salvia miltiorrhiza*, as well as their correlation with plant growth. The results showed that fertilization significantly affected the active ingredients and hormone content, soil physicochemical properties, and the composition of epiphytic microbial communities. After fertilization, the plant surface was enriched with a core microbial community mainly composed of bacteria from Firmicutes, Proteobacteria, and Actinobacteria, as well as fungi from Zygomycota and Ascomycota. Additionally, plant growth hormones were the principal factors leading to alterations in the epiphytic microbial community of *S. miltiorrhiza*. Thus, the most effective method of fertilization involved the application of base fertilizer in combination with foliar fertilizer. This study provides a new perspective for studying the correlation between microbial community function and the quality of *S. miltiorrhiza*, and also provides a theoretical basis for the cultivation and sustainable development of high-quality medicinal plants.

## 1 Introduction

Currently, artificial cultivation has become a necessity for the production of commercially cultivated medicinal plants, as it ensures consistent supply and quality, and allows for selective breeding to optimize medicinal properties (Pang et al., 2016; Niu et al., 2021). Fertilization is an essential approach for improving quality and efficiency in modern agriculture, and effective fertilization methods are key in determining the yield and quality of medicinal plants. Over-fertilization affects crop growth, leading to excessive enrichment of soil nutrients and a decrease in organic matter content, and causes soil acidification, salinization, and imbalances in microbial diversity. These present significant challenges to sustainable cultivation industries and seriously hinder the healthy development of the contemporary medicinal plant industry (Ti et al., 2015). Therefore, optimizing fertilization strategies, improving fertilizer utilization efficiency, and preventing soil degradation have received great attention (Geng et al., 2019; Iqbal et al., 2020; Ren et al., 2021; Shi et al., 2021). Foliar fertilization, as a supplement to plant nutrient absorption and compensation for insufficient root nutrient absorption, exhibits the characteristics of fast nutrient absorption, strong efficacy, low consumption, high efficiency, and reduced environmental pollution, making it an effective fertilization strategy (Kentelky and Szekely-Varga, 2021). In addition, foliar fertilization also affects the abundance and diversity of plant-associated microbial species (Lin et al., 2019). Studies have demonstrated that microorganisms can synthesize organic compounds, enzymes, hormones, and other bioactive substances, playing an important role in plant growth and development. Hence, further research on the effects of microorganisms on the growth of medicinal plants under the intervention of exogenous nutrients can help improve the yield and quality of medicinal plants, and promote the sustainable development of medicinal plant cultivation and production.

Studies have shown that microorganisms can enrich or colonize the surface or interior of plant tissues (Vorholt, 2012; Vandenkoornhuyse et al., 2015). Compared to endophytes, epiphytes naturally exhibit greater diversity in composition and abundance as they are directly exposed to different ecological environments (Chen et al., 2020). Typically, phyllosphere presents one of Earth’s most copious microbial habitats on its surface (Vorholt, 2012). These epiphytic microorganisms maintain a symbiotic relationship with host plants, significantly affect plant growth, immune defense and adaptation to environmental conditions (Berg, 2009; Berendsen et al., 2012). Importantly, the phyllosphere exists in a volatile and unstable environment in which the flora is subject to multifarious stresses, engendering a discernible trend and preference for certain microbial taxa (Bringel and Couée, 2015). Similarly, rhizospheric microorganisms are indispensable for plant ontogeny, engaging in a synergistic interaction with plant roots. These microorganisms aggregate around the roots, converting organic substrates into inorganic forms to provide vital nutrients for plants. In addition, they secrete factors that promote plant growth, including but not limited to vitamins and growth stimulants (Haney et al., 2015). Lu et al. (2018) found that rhizospheric microbes can modulate the timing of plant flowering, while recent reports have proposed the concept of microbial-root-upper ground, suggesting a profound interconnection between the subterranean and aerial plant components (Almario et al., 2017). Currently, researches on plant microbes have primarily focused on individual niches (Beckers et al., 2017), with limited studies reporting on changes in microbial community composition across different niches, ranging from the rhizosphere to the phyllosphere (Hacquard and SChadt, 2015). Fertilizer, as the most direct nutrient input, exert a significant impact on the epiphytic microbes in different ecological niches of plants, and the microbial community structure is also very sensitive to fertilization (Lozupone et al., 2012). Nutrients influence the community dynamics of pivotal microbial species, thereby regulating the composition of microbial communities (Schmidt et al., 2014). These key species are closely related to the nutrient cycle of carbon, nitrogen, and phosphorus within the soil, playing a vital role in enhancing crop productivity (Li et al., 2017a; Wang et al., 2022a).

*Salvia miltiorrhiza* Bge. (Labiaceae), a typical Chinese medicinal herb is widely used in the treatment of various diseases such as diabetes (Xie et al., 2021) and antiosteoporotic (Guo et al., 2014), with the efficacy in activating blood to eliminate stasis, transmissible pain relief, heart removal, and cool blood (Buja and Vander Heide, 2016). Research has demonstrated that fertilization and microbial interactions play a crucial role in determining the yield and quality of *S. miltiorrhiza*. For instance, Wei et al. (2023) reported that fertilization can significantly alter the rhizosphere microbial community, leading to improved biomass and medicinal quality of *S. miltiorrhiza*. Pu et al. (2022) found that different fertilization regimes can impact mycorrhizal symbiosis, thereby positively affecting the growth and active ingredient content of *S. miltiorrhiza*. Despite these findings, there are still gaps in the overall understanding of how the salvia quality is associated with the structure of the epiphytic microbial community under different fertilization strategies.

To investigate the influence of fertilization method and ecological niche variations on the composition of epiphytic bacterial and fungal communities in *S. miltiorrhiza* within field cultivation, and to explore the associations between epiphytes and their host plants, as well as the functional capabilities of epiphytes, we conducted a field experiment with three fertilization treatments (i.e., base fertilizer, foliar fertilizer, and base fertilizer+foliar fertilizer). The abundance, diversity, and composition of epiphytic bacterial and fungal communities in different ecological niches (leaves, stems, and roots) of *S. miltiorrhiza* were analyzed using Illumina Miseq high-throughput sequencing (HTS) technology. Additionally, co-occurrence networks of microorganisms were established to examine the interactions between fertilization and niche. We formulated the following hypotheses: (1) the composition of epiphytic microbial communities in *S. miltiorrhiza* varies depending on the fertilization method or ecological niches; (2) extensive intra-community interactions are expected to occur among epiphytes within the same niche or fertilization; (3) epiphytic microorganisms affect the growth of *S. miltiorrhiza* and rhizosphere soil properties. These findings will lay the groundwork for revealing the ecological functions of epiphytic communities in cultivation of medicinal plants, as well as the biodiversity and survival strategies of epiphytic communities under the condition of exogenous nutrition interference.

## 2 Materials and methods

### 2.1 Study site

The field experiment was conducted in Yaozhou District, Tongchuan City, Shanxi Province, China (34°48’–35°19’N, 108°34’–109°06’E) in the spring of 2022. The study area exhibits a characteristic temperate continental climate, characterized by an average annual temperature of 10.3°C, a precipitation level of 554.5 mm and a frost-free period of 217 d. Soil physicochemical characteristics were as follows: organic matter 0.22×10^−4^ mg/kg, total nitrogen 1246 mg/kg, available nitrogen 0.85×10^−5^ mg/g, available phosphorus 10.7762 mg/kg, and available potassium 0.1831×10^−3^ mg/kg.

### 2.2 Experimental design

A single-factor 4-level randomized block design was used to arrange the field experiment, with a plot area of 4 m × 4 m = 16 m^2^. Each fertilization treatment was set up in three replicates, a total of 4 × 3 = 12 plots, and a protection area of 350 m^2^ was set up around the experimental plot. The plant samples used in this study were one-year-old *S. miltiorrhiza* seedlings. Four fertilization treatments including base fertilizer (F1), foliar fertilizer (F2), base fertilizer +foliar fertilizer (F3) and blank control (CK) were applied. The base fertilizer was a compound fertilizer of nitrogen, phosphorus, and potassium from STANLEY from the local market, applied at a rate of 37.5 kg per acre. For foliar fertilizer, STANLEY potassium dihydrogen phosphate foliar fertilizer was used, with a spraying rate of 50–60 g per acre. Before cultivating seedlings, base fertilizer was applied to the soil, and then starting from the vigorous growth period of *S. miltiorrhiza* seedlings, spray foliar fertilizer three times every 15 d. Three ridges of 120 cm in width and 30 cm in height were set in each test plot, and a drainage ditch of 25 cm in width was set around the furrow. Two rows of *S. miltiorrhiza* were planted in each ridge with a row spacing of 15 cm×30 cm.

### 2.3 Collection of plants and soil samples

In March 2022, the experiment was planted and plant samples were collected at the end of the growing season in November 2022. Three healthy plants were randomly selected from each plot, and approximately 10 g of samples were collected from the leaves, stems, and roots using sterile surgical blades. Root samples for rhizosphere microbial analysis were placed in a 4°C cooler and subsequently brought back to the laboratory. Rhizosphere soil microbial analysis samples were from soil samples 10–15 cm away from the roots, and 200 g of soil sample was collected from each plot and placed in a sealed plastic bag. All soil samples were transported to the laboratory in insulated containers. Before experimenting, all samples were sieved (<2 mm mesh) to remove rocks, coarse roots and other litter. Soil samples used for enzyme analysis were stored in a 4°C refrigerator. Other soil subsamples were air-dried and used for determination of soil physicochemical properties. A total of 12 leaf, stem, root samples and 12 soil samples were collected. The samples for high-throughput sequencing were stored in a −80°C freezer for preservation.

### 2.4 DNA extraction, PCR and Illumina Miseq Sequencing

Five grams of plant sample (leaves, stems, or roots) were weighed and put into a 50 mL centrifuge tube, with 50 mL of 0.1 M potassium phosphate buffer (PPB, pH=8.0) added. Plant sample in tubes were washed with 1 min sonication and 10 s vortex, and repeated. Then the samples were transferred to new tubes with 50 mL of 0.1M PPB and washed again. The suspension from two washes was mixed and filtered through a 0.2 µm membrane. The filter membranes with epiphytes were snap frozen in liquid nitrogen and stored at −80°C in the refrigerator for subsequent DNA extraction (Bodenhausen et al., 2013).

The genomic DNA from epiphytic microorganisms was extracted from filter membranes using the FastDNA^®^ Spin Kit for Soil (MP Biomedicals, USA) according to the user’s manual. The DNA purity and concentration were measured with a NanoDrop 2000 spectrophotometer (Thermo Fisher Scientific, USA), and DNA integrity was examined using 1% agarose gel electrophoresis and stored at −20°C in a refrigerator for subsequent experiments.

The ABI GeneAmp^®^ 9700 PCR thermal cycler (ABI, USA) was used to amplify the 16S V3-V4 region (5’-GTGCCAGCMGCCGCGGTAA-3’ and 806R, 5’-GGACTACHVGGGTWTCTAAT-3’) of epiphytic bacteria and the ITS1 region (ITS1F/ITS2, 5’-CTTGGTCATTTAGAGGAAGTAA-3’ and 5’-GCTGCGTTCTTCATCGATGC-3’) of epiphytic fungi (Tamaki et al., 2011; Tepper and Gaynor, 2015). The PCR reactions were performed in a 20 μL system, including 2 μL of 10×buffer, 2 μL of 2.5 mM dNTP, 0.8 μL of each 5 μM primer, 0.2 μL of Taq polymerase, 0.2 μL of BSA, 10 ng of template DNA, and topped up to 20 μL with ddH_2_O. The amplification of the bacterial 16S V3-V4 region was carried out under the following conditions: denaturation at 95°C for 3 min; 30 cycles of 95°C for 30 s, 55°C for 30 s, 72°C for 45 s; and a final extension at 72°C for 10 min. For the fungal ITS1 region, PCR amplification was performed under the same reaction system and conditions, but with 35 cycles. Each amplification was repeated three times. The PCR products were recovered on a 2% agarose gel, and further purified using the AxyPrep DNA Gel Extraction Kit (Axygen Biosciences, Union City, CA, USA). Subsequently, the recovered PCR products were quantified using the Quantus™ Fluorometer (Promega, USA). The purified amplicons were mixed in equal amounts, and a library was constructed using the NEXTFLEX^®^ Rapid DNA-Seq Kit. The final sequencing was conducted using the Illumina MiSeq PE300 platform at Shanghai Majorbio Bio-pharm Technology Co., Ltd. The raw data have been deposited in the NCBI SRA database (PRJNA983712, PRJNA982701).

### 2.5 Bioinformatics analysis

Quality control for the raw reads was performed using software tools fastp (version 0.19.6) and FLASH (version 1.2. 11) with following steps: (1) filter bases with a mass value of less than 20 and read content containing N bases, set a window of 50 bp, and truncate bases if the average mass value in the window was less than 20, finally, the reading below 50 bp was filtered after quality control; (2) pairs of reads were spliced according to the overlap between PE reads, and the minimum length of overlap was 10 bp; (3) a maximum mismatch ratio of 0.2 was allowed in the overlap region of the spliced sequences, and inconsistent sequences were removed; (4) samples were demultiplexed based on the barcode. Quality-control splicing sequences were clustered into operational taxonomies (OTUs) based on 97% similarity using the UPARSE software (version 7.1). All sequences with mitochondrial and chloroplast annotations were removed. Taxonomic placement of epiphytic bacteria and fungi was annotated according to Silva 16S rRNA gene database (V 138) and UNITE database (version 8.0), respectively, based on an RDP classifier (version 2.11) with a 70% confidence threshold. The community composition of each sample was analyzed at various species taxonomic levels. Bacterial function prediction was based on the PICRUST2 database (Douglas et al., 2020), and bacterial function prediction was based on the FUNGuild database (Nguyen et al., 2016).

### 2.6 Measurement of plant biomass and morphological parameters

The weight of roots and stems was determined using an analytical balance. The lengths of the roots and shoots were ascertained using a tape measure. Plant water content was determined using the drying method. As described previously, the NBT photochemical reduction method was selected to test superoxide dismutase (SOD) activity (Cheng et al., 2015). Soluble protein content was determined using the Bradford assay (Kielkopf et al., 2020). Abscisic acid (ABA, ng/mL), cytokinin (CTA, ng/mL), gibberellin (GA, pmol/mL), Indole-3-acetic acid (IAA, nmol/L), and nitrate reductase (NR, µg/g.h) were measured using enzyme-linked immunosorbent assay (ELISA) detection kits.

### 2.7 Determination of active ingredient content

The medicinal ingredients of *S. miltiorrhiza* referred to in the 2020 edition of Chinese Pharmacopoeia I, and the lipid soluble chromatographic conditions were as follows: Silica gel bonded with octadecyl silane was used as the filler, acetonitrile was employed as mobile phase A, and a 0.05% phosphoric acid solution served as mobile phase B. The gradient elution conditions were indvided four parts: 0–6 min (A was 61% and B was 39%), 6–20 min (A changed from 61% to 90% while B changed from 39% to 10%), 20–20.5 min (A changed from 90% to 61% and B changed from 10% to 39%) and 20.5–25 min (A was 61% and B was 39%). The flow rate was maintained at 1 mL/min. The fat-soluble components determined included cryptotanshione, Tanshinone I, and Tanshinone IIA. Water soluble chromatographic conditions were as follows: using a C_18_ chromatographic column, acetonitrile was employed as mobile phase A, and a 0.05% phosphoric acid solution served as mobile phase B. The four parts of gradient elution for 0–15 min, 15–30 min, 30–40 min and 20.5–25 min were A of 17–23%, 23–25%, 25–90%, and 90% and B of 83–77%, 77–75%, 75–10%, and 10%, respectively. The column temperature was maintained at 30°C with a flow rate of 1 mL/min at a detection wavelength of 286 nm. The water-soluble components determined included salvianolic acid B and rosmarinic acid.

### 2.8 Determination of soil parameters

Soil pH was measured in a 1:2.5 (w/w) soil: water suspension with pH 3000 (STEP Systems Gmbh, Germany). Soil organic carbon (SOC) was determined using the combustion loss method (Heiri et al., 2001). The available phosphorus in soil (AP) was measured using the sodium bicarbonate extraction-molybdenum antimony colorimetric method (Olsen et al., 1954), with 0.5 mol/L sodium bicarbonate solution used for extracting the available phosphorus, which reacted with molybdenum antimony to generate phosphomolybdenum blue. The available potassium (OP) was determined using the tetraphenylboron method (Chen et al., 2019), with 1 mol/L NaNO_3_ solution used for extracting soil K^+^, which reacted with tetraphenylboron in a weakly alkaline medium to produce a barely soluble white precipitate. The measurement of ammonium nitrogen (NH_4_^+^-N), nitrate nitrogen (NO_3_^−^-N), total phosphorus (TP), and total nitrogen (TN) was performed using the Smartchem 200 analyzer (Alliance, France) (Xie et al., 2017). The activity of sucrase (SC) was determined using the method of Guan (1986). The sucrase in soil catalyzes the hydrolysis of sucrose into reducing sugar, which reacts with 3,5-dinitrosalicylic acid to produce orange 3-amino-5-nitrosalicylic acid under the boiling condition, and the depth of color is positively correlated with the content of reducing sugar. The measurement of nitrate reductase (NR) activity was carried out using the sulfanilamide diazotization colorimetric method (Zhao et al., 2009), where the reaction of nitrite with sulfanilamide and α-naphthylamine under acidic conditions produces a red compound. The urease (URE) activity was measured using the improved Hoffmann and Teicher colorimetric method (Kandeler and Gerber, 1988), with urea as the substrate, and indophenol, which is generated by the reaction of enzyme products and phenol-sodium hypochlorite, is used for analyzing the activity of urease. The activity of alkaline phosphatase (ALP) was measured using the method described by Tarafdar and Marschner (1994), with disodium phenyl phosphate (pNPP) as the substrate. The substrate is hydrolyzed by soil acid phosphatase to produce yellow p-nitrophenol (pNP), and the amount of pNP produced is directly proportional to the absorbance of the yellow solution, which can be used for quantitative analysis. The data analysis was performed using SPSS software version 24.0 (IBM SPSS Statistics, USA).

### 2.9 Statistical analysis

Alpha diversity (Shannon, Chao1, goods_coverage, and pielou_e indices) and beta diversity analysis (NMDS) were computed using the QIIME2 software. Statistical analysis and plotting for Mantel tests were performed using three R packages (dplyr, linkKEt, and ggplot2; http://www.R-project.org). Bivariate correlations between plant growth parameters, soil physicochemical properties, and microbial communities were analyzed using SPSS 24.0 software, with Tukey’s test used to compare means (*P*<0.05). Variance partition analysis (VPA) was used to investigate the effects of growth parameters, soil properties, and plant hormones on the microbial abundance in different parts of Salvia miltiorrhiza. Pheatmap and Vegan were used for statistical analysis and data visualization. Network parameter analyses were conducted using six R packages (igraph, psych, Hmisc, vegan, dplyr, and reshape2). The βNTI value was calculated to assess community assembly processes, using the formula below (Equation 1):


(1) $$\text{βNTI}=\frac{{\text{βMNTD}}_{\text{obs}}-\bar{{\text{βMNTD}}_{\text{null}}}}{sd{\text{βMNTD}}_{\text{null}}}$$

## 3 Results

### 3.1 Composition of the epiphytic microbial community

The regression curves of OTUs observed at the OTU level for *S. miltiorrhiza* epiphytic bacteria and fungi gradually flattened, indicating that the amount of sequencing data for epibiotic bacteria and fungi was reasonable and the OTU depth met the requirements for diversity analysis (**Supplementary Figure S1**). A total of 4256 OTUs of epiphytic bacteria were obtained, belonging to 361 species, 544 genera, 146 orders, 251 families, 53 classes and 27 phyla. In addition, 1702 OTUs of epiphytic fungi were identified, belonging to 299 species, 226 genera, 123 families, 56 orders, 23 classes, and 8 phyla.

At the phylum level (**Supplementary Figure S2A-a**), Actinomycetes and Firmicutes were the dominant groups of epiphytic bacteria in *S. miltiorrhiza* leaves, with the abundance of Actinomycetes (13.25%, 8.42%) and firmicutes (13.25%, 8.42%), respectively. The relative representation of Firmicutes (10.22%) and Bacteroidetes within the root system exhibited a reduction, whereas the prevalence of Proteobacteria (75.77%) experienced an elevation. Interestingly, Acidobacteriota expression decreased under both fertilization treatments (**Supplementary Table S1**). At the genus level (**Supplementary Figure S2A-b**), a decline in the prevalence of *Ralstonia* and *Massilia* was observed across both fertilization treatments. The concentration of *Hymenobacter* (8.21%, 9.26%), *Rhizobium* (7.53%), and *Sphingomonas* (8.97%) also increased. The proportion of *Pseudomonas* was elevated to 19.14% and 13.82% in the group F2 in leaves, heightened in the groups F2 and F3 in stems, rose in the group F1 in roots, and reduced in the other treatment groups. At the phylum and genus level (**Supplementary Figures S2A-c, d**), fertilization treatments had a lesser effect on altering the abundance of the epibiotic fungal community in *S. miltiorrhiza*.

After the fertilization treatment, the unique bacterial OTUs of the three parts of *S. miltiorrhiza* were lower than those of the blank treatment except for the YF1 treatment (**Supplementary Figure S2B-a**), and the number of bacterial OTUs was curtailed after the fertilization treatment. The most common bacterial OTUs were found in roots. The number of unique fungal OTUs in the three parts of *S. miltiorrhiza* increased as a whole except for a few (**Supplementary Figure S2B-d**). The combination of foliar fertilizer plus base fertilizer showed the greatest increase in OTUs, doubling the amount on average in all three sites. The leaves had the most common fungal OTUs, and the number of bacterial and fungal OTUs decreased under the combination of leaf fertilizer and basal fertilizer.

### 3.2 Analysis of α-diversity of epiphytic communities

**Figure 1:**
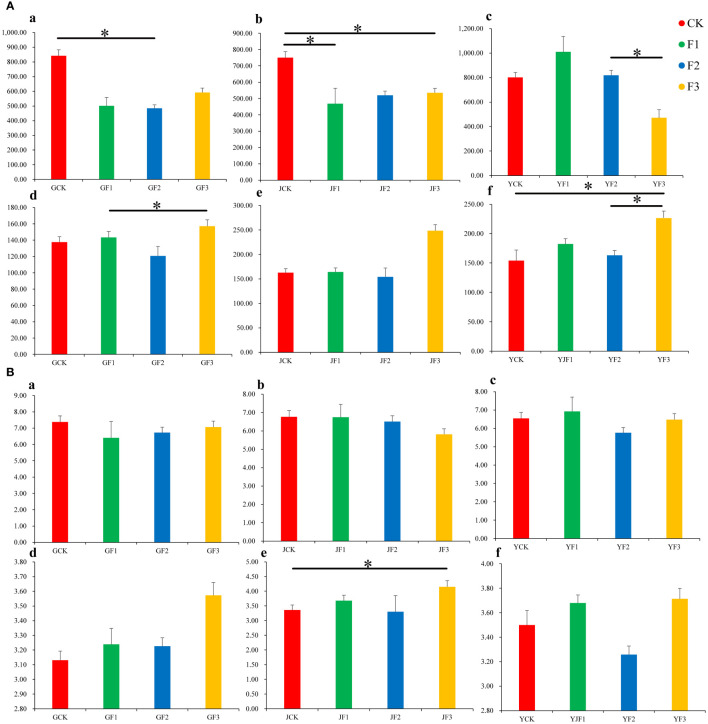
The alpha diversity of bacterial and fungal OTUs in the roots, stems, and leaves of *Salvia miltiorrhiza*. a–c are bacteria. d–f are fungi. **(A)**, chao1. **(B)**, Shannon * *P*<0.05. Y, leaf. J, stem. G, root.

When evaluating the α-diversity of epibiotic bacteria in different parts of *S. miltiorrhiza* based on the OTU level we found that the Good’s coverage index values were all close to 1, indicating that the sequencing depth was qualified. This further indicated a negligible likelihood of undetected sequences across the analyzed samples (**Supplementary Table S2**). The Shannon index and Chao1 index of bacteria and fungi were tested by T-test. After fertilization, no significant change was found the diversity of epiphytic bacteria and fungi from in the three parts of *S. miltiorrhiza*, but there was a substantial variation in their abundance (**Figure 1**). Different fertilization treatments had varying effects on abundance. For example, in the leaf section, the concentration of bacteria and fungi was significantly higher in groups F2 and F3 than in group CK. This pattern was also observed in the root section, while only bacteria showed a difference in the stem section. Under fertilization treatment, the diversity of epiphytic bacteria and fungi (excluding stem-associated bacteria) had undergone alterations across groups, albeit not apparently, namely there was a reduction in bacterial diversity and an increase in fungal diversity. Excluding stem bacteria, pronounced fluctuations in microbial community proportions were evident, with the F3 treatment cohort exhibiting the most marked alterations, typified by diminished bacterial and heightened fungal levels.

### 3.3 Comparative analysis of the similarity of epiphytic communities

**Figure 2:**
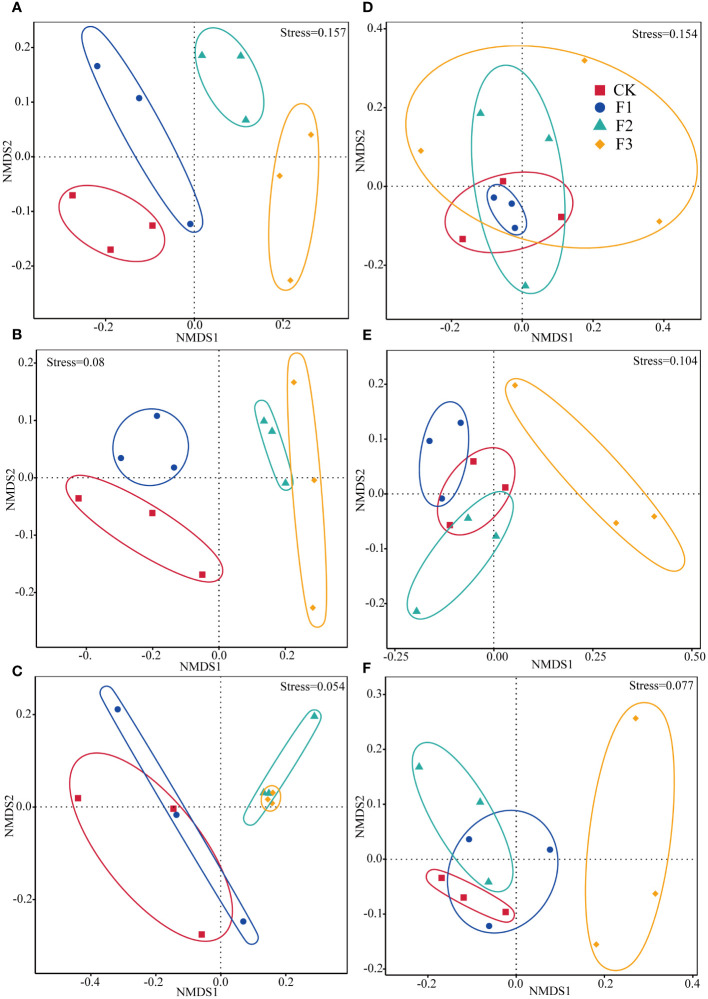
The non-metric multidimensional scaling (NMDS) ordination of bacterial and fungal community composition in the roots, stems, and leaves of *Salvia miltiorrhiza*. Base fertilizer (F1), Foliar fertilizer (F2), Base fertilizer +foliar fertilizer (F3), Blank control (CK). Root **(A, D)**. Stem **(B, E)**. Leaf **(C, F)**. **(A–C)** are bacteria. **(D–F)** are fungi.

The NMDS and ANOSIM tests indicated that there was no salient dissemblance in the bacterial and fungal community structure on different parts of *S. miltiorrhiza*, and the same phenomenon was observed among treatment groups. (**Supplementary Table S3**; **Figure 2**). Bacterial community similarity analyses revealed divergent structure compositions in leaves between F2 and F3 cohorts versus groups CK and F1. Analogous differentiation patterns were noted across stem-based bacterial populations. Stem-based bacterial community structures exhibited variability between groups F2 and CK. In the roots, all treatments showed divergent community structures (**Figures 2A–C**). For fungal communities, the community structure of the F3 treatment group was distinct from the CK, F2 and F1 treatments. The fungal community composition in the F2 treatment diverged from the control (CK). In stem, the communities within the F3 treatment were distinct compared to those in the CK, F2, and F1. Community structure in the F1 exhibited variations from the CK. The community structures of epiphytic fungi and bacteria across the three parts of rhizome and leaves were different but these changes were not prominent.

### 3.4 The construction process of epiphytic communities

**Figure 3:**
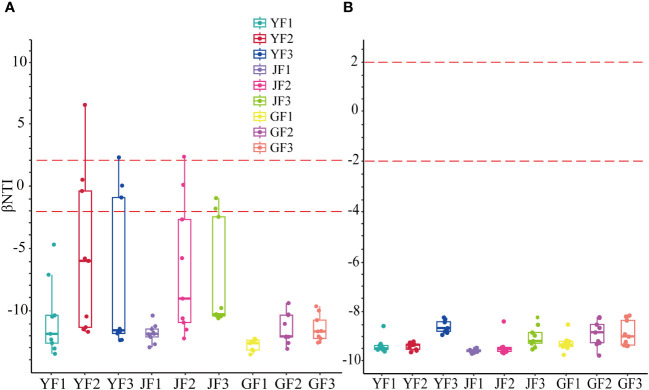
The null model analysis of bacterial **(A)** and fungal **(B)** communities in the roots, stems, and leaves of *Salvia miltiorrhiza* based on βNTI. Y, leaf. J, stem. G, root.

Based on the zero βNTI system diversity model analysis, the results showed that in *S. miltiorrhiza* (root, stem, and leaf), at different positions of the surface, bacterial and fungal microbial communities were built in a largely deterministic process (defined as β|NTI| > 2) (Dini-Andreote et al., 2015) (**Figure 3**). This choice was mainly homogeneous. In fungal communities, all three fertilization methods converted the microbial construction process into a deterministic one. Among the bacterial communities, group F1 had the greatest impact on the construction process compared to the other two treatments, and roots were the most sensitive to the construction process.

### 3.5 Co-occurrence network of epiphytic microorganisms

**Figure 4:**
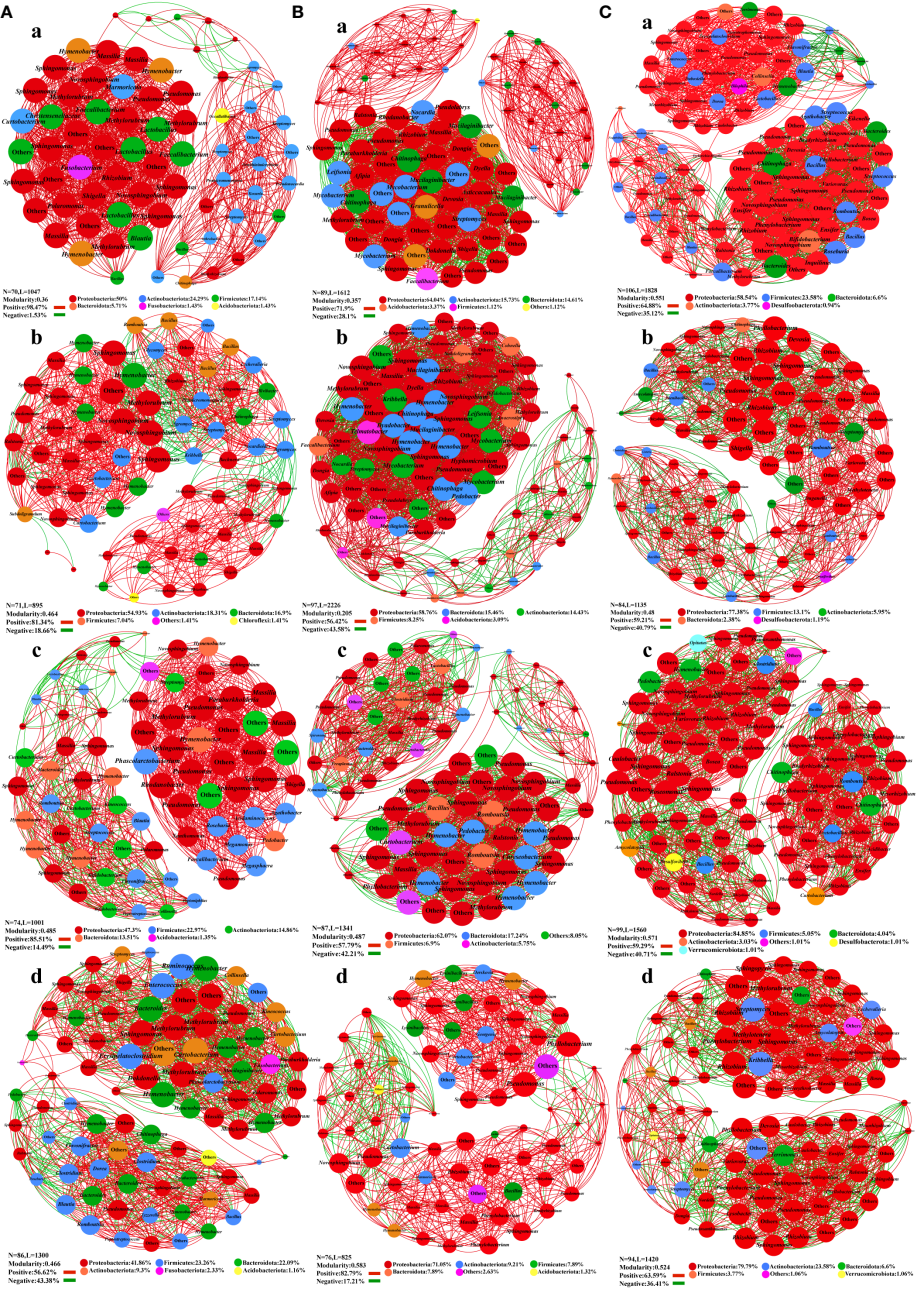
Co-occurrence network plots analysis of the bacterial community structure in *Salvia miltiorrhiza* under different fertilization treatments. **(A)**, Leaf. **(B)**, stem. **(C)**, roots. a, CK treatment group. b, F1 treatment group. c, F2 treatment group. d, F3 treatment group.

**Figure 5:**
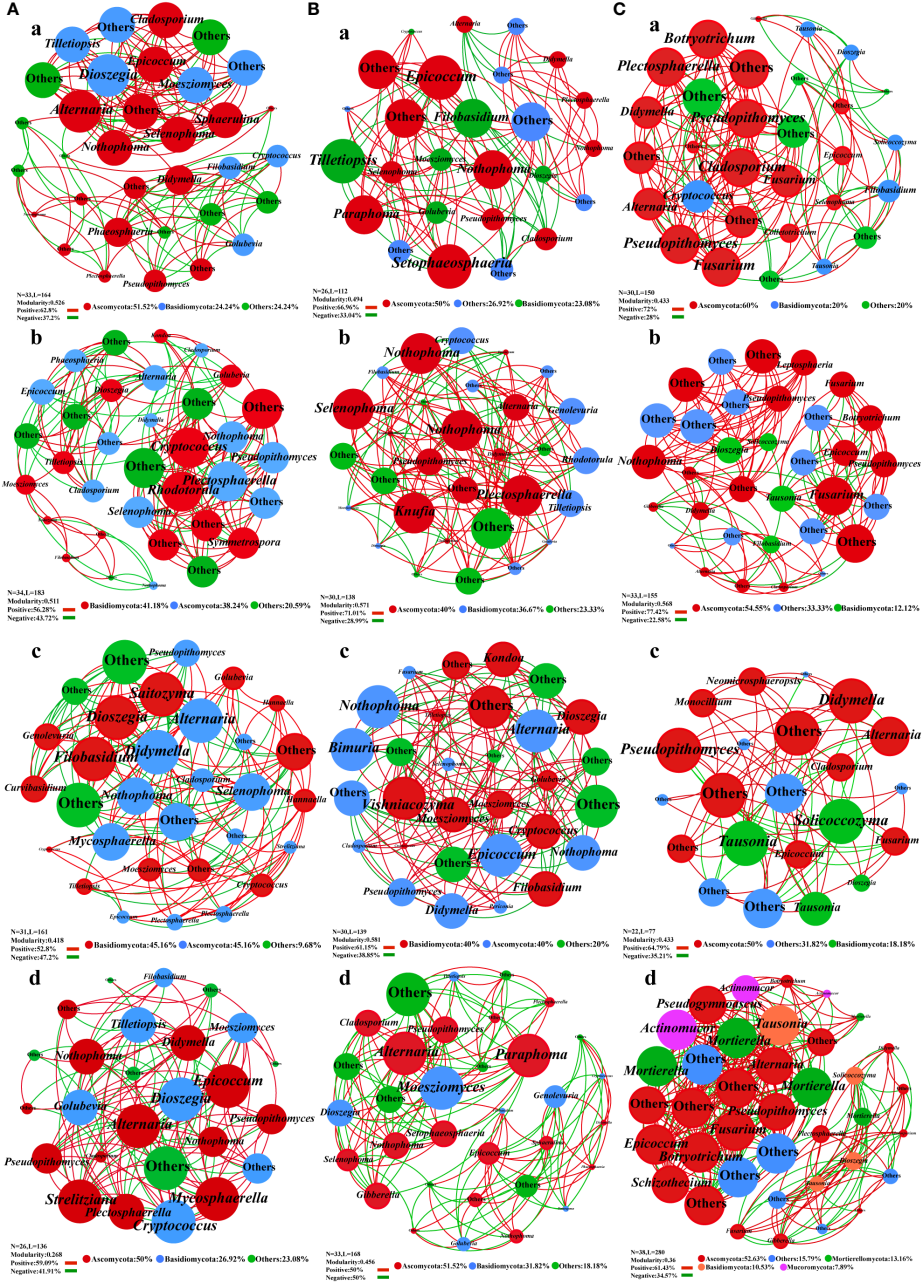
Co-occurrence network analysis of fungal community structure in *Salvia miltiorrhiza* under different fertilization treatments. **(A)**, Leaf. **(B)**, stem. **(C)**, roots. a, CK treatment group. b, F1 treatment group. c, F2 treatment group. d, F3 treatment group.

A co-occurrence network map was constructed with the measured OTUs to illustrate the general symbiosis model of bacteria (**Figure 4**) and fungi (**Figure 5**) under different fertilization treatments for the key species group of epiphytic bacteria. In the leaf bacterial network, Bacteroidota and *Hymenobacter* were significantly increased in the F1 compared with the CK. Firmicutes increased in F2 and F3, and the composition of the major genus-level nodes in this phylum was no longer dominated by *Lactobacillus* and *Faecalibacterium*, but switched to other genera (**Figure 4A**). Moreover, the modularity index and the number of nodes increased after fertilization, and the proportion of positive correlation decreased, which might make the community structure more stable. In the stem bacterial network, compared to the CK treatment, the F1 had more nodes and edges, but the modularity index was reduced. It can also be seen from **Figure 4B** that most nodes were clustered together, and the distribution was not uniform. For groups F3 and F2, the node distribution was more uniform, meaning that even though the number of nodes and edges decreased, the modularity index was increased, thereby suggesting that the bacterial community in the stem may be more stable after the application of foliate fertilizer. In the root bacterial network, the community structure of roots was more stable than that of leaves and stems, and the modularity index of all treatment groups was greater than 0.4. The positive and negative relationships among species and the node distribution were also more uniform (**Figure 4C**). There were no significant differences between the treatment groups, but the number of nodes and edges in the network decreased after fertilization.

In the leaf fungal network, compared with CK treatment, Basidiomycota was the dominant phylum in groups F1 and F2, and *Cryptococcus* and *Rhodotorula* were the leading genera in groups F1 and F2. In the CK, *Dioszegia*, *Moesziomyces* and *Tilletiopsis* were the main genera. Ascomycota was the principal phylum in CK and F3, while *Alternaria* and *Epicoccum* were the major genera in CK and F3, and *Mycosphaerella* was added in F3 (**Figure 5A**). Fertilization treatment gradually reduced the modularity index of leaf epiphytic fungi and the stability of the network. In the stem fungal network, compared to the CK treatment, the F2 treatment group was dominated by Basidiomycota, and the species at the genus level had changed greatly. The F2 treatment group was mainly controlled by *Vishniacozyma* and *Cryptococcus*. Moreover, *Tilletiopsis* was the primary species in the CK treatment. Ascomycota was the prevailing phylum in groups CK, F1, and F3. *Setophaeosphaeria* and *Epicoccum* were the superior species in CK. The genera *Plectosphaerella*, *Knufia*, and *Selenophoma* were prevalent in the F1, and *Alternaria* and *Paraphoma* were predominant genera in the F3 (**Figure 5B**). Importantly, fertilizer treatment also changed the composition of fungal communities in the stems. In the root fungal network, compared to group CK, the distribution of nodes in F1 was more uniform, the number of nodes and edges in F2 was reduced, and the modularity index of the two treatments was greater than 0.4. Additionally, Mucoromycota and Mortierellomycota were added as new phyla to the F3. But the modularity index was less than 0.4 (**Figure 5C**).

### 3.6 Prediction of function

PICRUST2 database was used to predict the function of the bacterial community under different fertilization treatments (**Supplementary Figure S3-a**). It is remarking that the protein function was higher in leaves than in other parts. For instance, the protein function of glycosyltransferases involved in cell wall biosynthesis, as well as the acyl coenzyme A dehydrogenase associated with the alkylation reaction protein AidB, were significantly enhanced. These functions play a pivotal role in plant development and stress response.

The function of epibiotic under different fertilization treatments was determined by the The FUNGuild database, and the result was plotted in **Supplementary Figure S3-b**. The predicted fungal functions mainly include three categories: pathogenic, symbiotic, and saprophytic. It is subdivided into animal pathogens, arbuscular mycorrhizal fungi, ectomycorrhizal fungi, lichenized fungi, mycoparasites, plant pathogens, undefined saprotrophs, and wood saprotrophs. Animal pathogens, plant pathogens, and undefined saprophytic fungi were saliently enhanced.

### 3.7 Plant growth parameters

**Figure 6:**
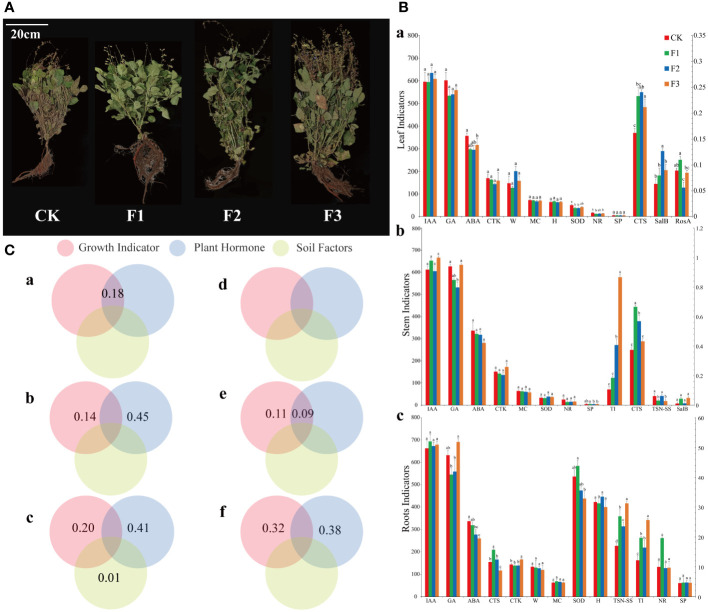
*Salvia miltiorrhiza* growth plot, bar plot of growth parameters, variance decomposition plot of growth parameters and hormones with microbial community. **(A)**, CK: blank control, F1: basal fertilizer, F2 foliar fertilizer, F3 basal fertilizer plus foliar fertilizer. **(B)**, a: leaf, b: stem, c: Root, leaf length (B, a, H), leaf weight (B, a, W), root length (B, c, H), root weight (B, c, W), leaf water content (MC), plant superoxide dismutase (SOD), soluble protein (SP), abscisic acid (ABA), cytokinin (CTK), gibberellin (GA), auxin (IAA), nitrate reductase (NR), tanshinone II A (TSN-SS), tanshinone I (TI), cryptotanshinone (CTS), salvianolic acid B (SalB), and rosmarinic acid (RosA). (The presence of identical superscript letters indicates a nonsignificant difference, while distinct superscript letters denote statistical significance) **(C)**, a–c refers to bacteria, d–f to fungi, a, d to leaves, b, e to stems, and c, f to roots.

The results revealed significant disparities in the growth parameters of the leaves, stems, and roots following fertilization. (**Figures 6A, B**). There was a reduction in the levels of abscisic acid (ABA), superoxide dismutase (SOD) and nitrate reductase (NR), while the levels of Cryptotanshinone (CTS), rosmarinic acid (RosA) and salvianolic acid B (SalB) increased, The gibberellin (GA), soluble protein (SP), and tanshinone IIA (TSN-SS) content decreased in the stems, while the content of tanshinone I (TI) and RosA increased. The concentration of GA, ABA and CTS decreased in the roots, but the content of liposoluble medicinal ingredients increased. The comparison among different treatment groups revealed that the impact of group F3 was the most pronounced.

Analysis of the ANOVA (**Figure 6C**) showed that leaf growth parameters and hormones together explained 18% of the variation in the epibiotic bacterial community but without the fungal variation. For the stem, growth parameters and hormones accounted for 14% and 45% of the bacterial community variation, respectively, and were the main factors affecting the bacterial community. 11% of the variation in fungal communities can be attributed to growth parameters, but the combined effects of growth parameters and hormones can only cause 9% of changes. In terms of roots, growth parameters and hormones were the main factors affecting the bacterial community, and the variation in the bacterial community can reach 20% and 41%, respectively. Variations in the fungal community are predominantly governed by growth parameters and hormonal activity, contributing to 32% and 38% of the modifications, correspondingly.

### 3.8 Mantel association analysis of epiphytic communities

#### 3.8.1 Association analysis between epiphytic communities and growth parameters

**Figure 7:**
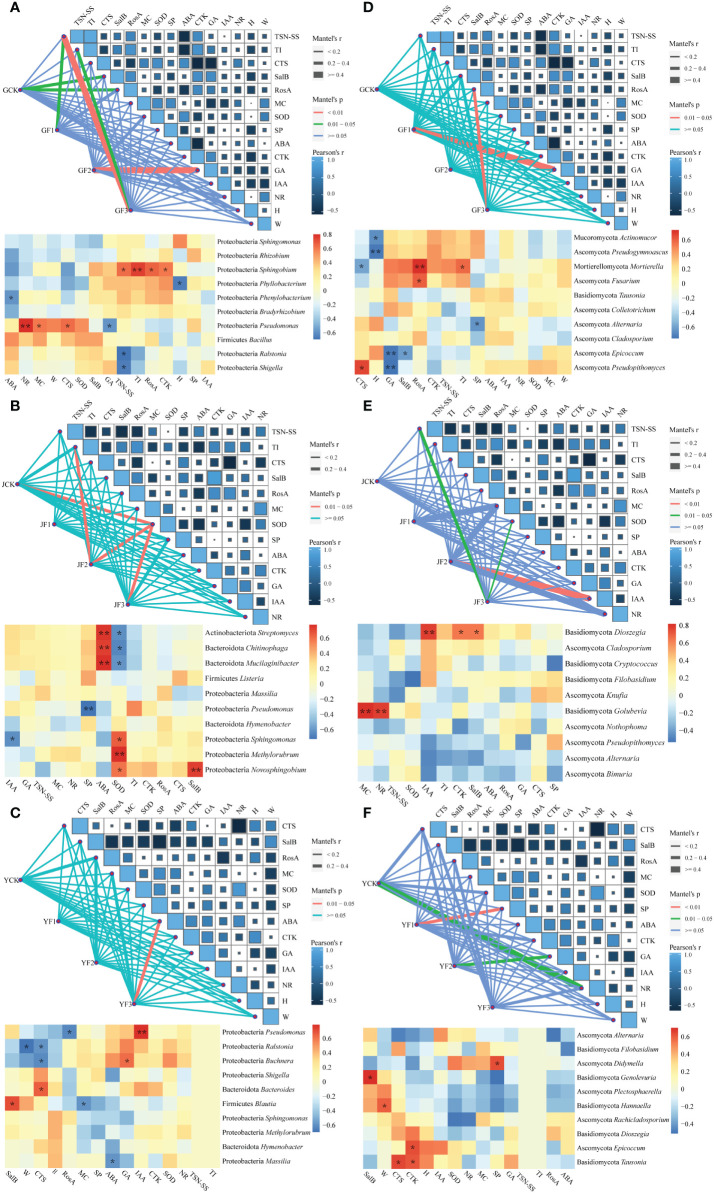
Mantel analysis map and genus-level correlation heat map of community structure, function, and growth parameters of bacterial (a, b, c) and fungal (d, e, f) communities in the root **(A, D)**, stem **(B, E)** and leaf **(C, F)** of *Salvia miltiorrhiza.* Leaf length (c, f, H), leaf weight (c, f, W), root length (a, d, H), root weight (a, d, W), leaf water content (MC), plant superoxide dismutase (SOD), soluble protein (SP), abscisic acid (ABA), cytokinin (CTK), gibberellin (GA), auxin (IAA), nitrate reductase (NR), tanshinone II A (TSN-SS), tanshinone I (TI), cryptotanshinone (CTS), Salvianolic acid B (SalB), and rosmarinic acid (RosA). *P< 0.05, **P< 0.01. Y, leaf. J, stem. G, root.

The Mantel analysis revealed that after fertilization, GA manifestly impacted the community structure of root surface bacteria and fungi. Medicinal components such as TSN-SS, TI, and RosA were found to be associated with changes in the root surface microbiota. (**Figure 7**). The alterations in stem SOD affected the epiphytic bacteria, whereas IAA influenced the epiphytic fungi. The TSN-SS and TI in the stem were closely linked to the changes in epiphytic microbiota. The growth parameters had a minimal impact on the structure of the epiphytic bacterial microbiota in the leaves, but notably affected the epiphytic fungi, especially GA and SP. In the context of the three fertilization methods, it was anticipated that group F3 would outperform the other treatment groups. The effects of F3 on microorganisms and growth parameters were expected to be more significant compared to the other two treatments.

We conducted a correlation analysis between growth parameters and the top ten microorganisms in terms of relative abundance at the genus level. We found that the growth parameters markedly influenced the presence of Proteobacteria, Mucoromycota, Ascomycota and Mortierellomycota in the roots. The key genera that were beneficial for medicinal compounds and hormones included *Sphingobium*, *Mortierella*, and *Fusarium*. The growth parameters exhibited mutual influences on the stem Bacteroidota, Actinobacteriota, Proteobacteria and Basidiomycota. Among these, the core genera with positive effects included *Novosphingobium* and *Dioszegia*. The leaf microbiota included Proteobacteria, Bacteroidota, Firmicutes, Basidiomycota and Ascomycota, with core genera that exerted positive influences, including *Blautia* and *Genolevuria*.

#### 3.8.2 Association analysis between epiphytic communities and environmental factors

**Figure 8:**
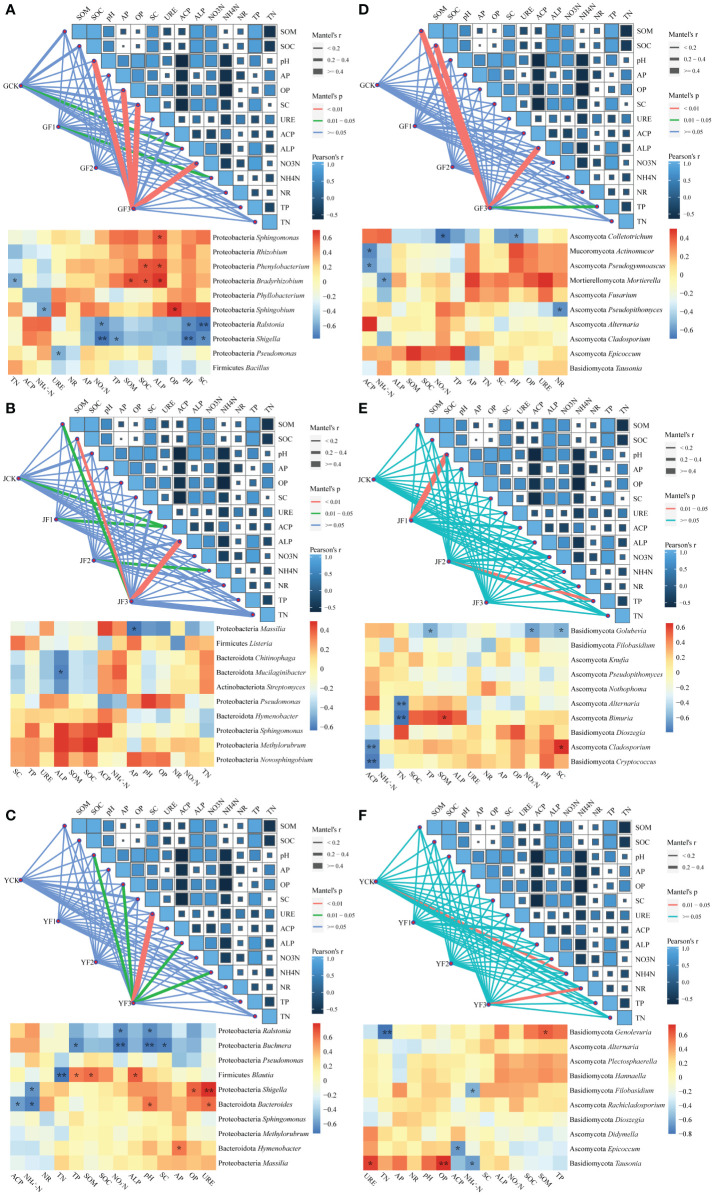
Mantel analysis map and genus level heat map of association between community structure and function of surface bacteria **(A–C)** and fungi **(D–F)** in the root **(A, D)**, stem **(B, E)**, leaf **(C, F)** and environmental factors of *Salvia miltiorrhiza*. Available phosphorus (AP), available potassium (OP), alkaline phosphatase (ALP), ammonium nitrogen (NH_4_^+^-N), acid phosphatase (ACP), urease (URE), nitrate reductase (NR), organic matter (SOM), organic carbon (SOC), sucrase (SC), nitrate nitrogen (NO_3_^−^-N), total phosphorus (TP), total nitrogen (TN). *P< 0.05, **P< 0.01. Y, leaf. J, stem. G, root.

Performing Mantel analysis on the soil environment and epiphytic microbiota of *S. miltiorrhiza*, we discovered a substantial influence of environmental factors on the response of root-associated epiphytic bacteria following fertilization, encompassing NH_4_^+^-N, pH, OP, SC, and NO_3_^−^-N (**Figure 8**). The stem section exhibited no correlation, while the epiphytic bacteria on the leaf surface showed correlations with pH, OP, URE, ALP, and NH_4_^+^-N. After fertilization, environmental factors had a greater impact on epiphytic bacteria than on epiphytic fungi, and only the root and leaf sections showed a response. Similarly, group F3 in the three fertilization methods was expected to exert a more pronounced influence on environmental factors and microorganisms.

In examining the relationships between microbe and environmental factors, the Firmicutes, Proteobacteria, Mucoromycota and Ascomycota were identified in the root section and exhibited noteworthy correlations with environmental factors. Notably, among these, the genera *Sphingomonas*, *Phenylobacterium*, *Bradyrhizobium*, and *Sphingobium* demonstrated a positive correlation with the soil factors. The core genera in the stem section did not exhibit a positive correlation with environmental factors. However, in the leaf section, Proteobacteria, Bacteroidota, Ascomycota and Basidiomycota were found to be correlated. The core genera within these were *Buchnera*, *Blautia*, *Shigella*, *Bacteroides*, *Hymenobacter*, *Genolevuria*, and *Epicoccum*.

## 4 Discussion

### 4.1 Effects of fertilization methods on the epiphytic community communities in different niches of *S. miltiorrhiza*

Many studies have shown that reasonable combined application of fertilizers can improve crop yield and quality. For example, for tea, fertilizers elevated the content of amino acids and tea polyphenols while reducing the phenol-to-ammonia ratio (Liu et al., 2023a). Similarly, cucumber yield and vitamin C content were improved with fertilizer application (Wang et al., 2023). In apples, fertilizers increased yield, soluble sugar and vitamin C content, resulting in a higher sugar–acid ratio (Wang et al., 2022b). In this study, plant growth parameters, biomass and the levels of active components were increased, indicating that fertilization could improve the quality of *S. miltiorrhiza*. Importantly, the effect of the F3 treatment was the most significant.

Recent investigations have shown that the structure of microbial communities is sensitive to fertilization (Lozupone et al., 2012). That is, nutrients can evidently affect the community changes of key microbial species and ultimately regulate the construction of microbial communities (Schmidt et al., 2014). These keystone species are closely related to the nutrient cycling process of carbon, nitrogen, and phosphorus in soil and play a considerable role in improving crop productivity (Li et al., 2017b). The analyzed results from the βNTI model showed that the composition of bacterial and fungal communities in different parts of *S. miltiorrhiza* (roots, stems, leaves) was a deterministic process. As an abiotic factor, fertilization determined the microbial community, which had a significant effect on the composition of bacterial and fungal communities in *S. miltiorrhiza*. Jiao and Lu (2020) analyzed soil fungal communities in farmland, forest, wetland, grassland, and desert ecosystems in the Hexi Corridor of China and found that the communities of rare taxa were mainly regulated by deterministic processes of homogeneous selection. Similarly, Xiong et al. (2021) studied fungal community structure in soil, root surfaces and leaf surfaces under different fertilization practices in maize/wheat rotation and maize/barley rotation field systems in different places and found that fungal community structure was mainly affected by deterministic processes. Although only basal fertilizer was applied, the microbial community construction in the shoot was still a deterministic process, indicating a strong connection between the belowground section of plants and the shoot.

Recently, the concept of microbial-root-shoot was also proposed (Almario et al., 2017). In the fungal communities, all three fertilization methods made the microbial construction process a deterministic one in our study. Among the bacterial communities, the F1 treatment had the greatest impact on the construction process compared to the other two treatments, and the root was the most sensitive to the construction process. The different responses of these bacteria and fungi may stem from changes in community stability, as well as varying sensitivities to fertilizers among fungi and bacteria. The results showed that the selection process of the construction of the epiphytic communities of *S. miltiorrhiza* was distinctly affected by fertilization. Fertilization had a filtering effect on the species of epiphytic communities of *S. miltiorrhiza*, which directly affected the survival of the epiphytic communities. Moreover, the selection was mainly homogeneous, and the similar abiotic environments such as fertilization interact with the surface microorganisms of *S. miltiorrhiza* to form the selection effect. Combined current studies show that fertilization can evidently affect microbial community construction, markedly impact the composition and abundance of key species (Lin et al., 2019), and drive changes in microbial community structure and ecosystem function (Fan et al., 2019; Han et al., 2022).

### 4.2 Effects of fertilization methods on the key species of epiphytic communities in different niches of *S. miltiorrhiza*

It is proved that the fertilization can significantly change plant microbial community composition and diversity (Sabir et al., 2021; Guan et al., 2022; Zhang et al., 2022). An increase in nutrient availability correlates with accelerated microbial growth rates and enhanced substrate metabolism (McCann et al., 1998). Fertilizers are even shown to impact light competition after eutrophication (Hautier et al., 2009), which leads to a reduction in plant microbial diversity. In this study, the abundance of the dominant taxa and genus groups of the epimicroorganisms in different ecological niches of *S. miltiorrhiza* decreased after fertilization, and the changes of fungi were reduced compared to bacterial communities, which may be due to the different establishment methods of fungal and bacterial community structure. Among them, in comparison to the other treatment groups, the effect of F3 was significantly stronger. These results indicated that fertilization greatly affected the composition and abundance of vital species in the epimicrobial community of *S. miltiorrhiza* (Lin et al., 2019). Data from diversity index of α, NMDS, and ANOSIM tests showed that the diversity of bacterial and fungal flora in different parts of *S. miltiorrhiza* was altered but there was no meaningful difference between the groups, while the abundance of bacterial flora was markedly different. The findings indicated that fertilization does not exert a substantial impact on the diversity of *S. miltiorrhiza* epiflora. The lack of this effect may be attributed to the complexity of the epiphytic environment, where there are multiple factors that affect microbial colonization, and fertilization is not the main determining factor (Bringel and Couée, 2015). Despite the absence of statistical significance, modifications in certain pivotal species were observed, warranting additional investigation into the alterations of these key species.

Microbes live in complex communities (Ley et al., 2006; Fuhrman, 2009; Faust and Raes, 2012). In addition to abiotic factors affecting microbial diversity, interactions among organisms also play an important role in the overall composition, stability, and biodiversity of microbial ecosystems (May, 1988; Wardle, 2006; Mougi and Kondoh, 2012). They can compete for resources, inhibit the growth of other communities by producing antibiotics, or support each other by cross-feeding (Ives and Carpenter, 2007; Ptacnik et al., 2008; Pande et al., 2014). In general, higher biodiversity often (but not always) implies greater ecosystem stability (Hector et al., 1999). The structure and diversity of microbial communities are sensitive to external environmental factors such as fertilization and irrigation (Lozupone et al., 2012; Bhattacharyya et al., 2017; Chen et al., 2020; Dangi et al., 2020; Khan et al., 2021). That is, nutrients affect the community changes of key microbial species and ultimately regulate the construction of microbial communities. These keystone species are closely related to the nutrient cycling process of carbon, nitrogen, and phosphorus in soil, and have a significant effect on improving crop productivity (Li et al., 2017b).

The node, edge, node type, positive correlation, and modularity index of the co-occurrence network were all increased after different fertilization treatments, indicating that community structure became more stable. The root and stem network had an increased abundance of Proteobacteria, a nutrition-sensitive phylum that include a variety of pathogenic bacteria (Dai et al., 2018). Acidobacteria species decreased in leaf sites, and Acidobacteria abundance was negatively correlated with nutrient content (Fierer et al., 2007; Banerjee et al., 2016), which can promote the dissolution of soil inorganic P, and their abundance was significantly correlated with soil available P (Flieder et al., 2021; Wu et al., 2021). Increased root *Rhizobia*, microorganisms that favor crop–microbe interactions, usually perform beneficial functions for the host by providing a variety of nutrients and metabolites (Erlacher et al., 2015; Gazdag et al., 2018; Chang et al., 2022). Ascomycota was the most abundant phylum in the fungal network, and fertilization promoted the growth of Ascomycota (Guo et al., 2020). After fertilization, *Cryptococcus*, *Rhodotorula*, *Apicophorus*, and *Alternaria* were widely distributed in the fungal network structure. The structure of group F3 was more stable among different treatments, mainly origin the combination of two kinds of fertilization resulted in more sufficient and comprehensive fertility across the different parts of the plant. The results showed that fertilization treatment can change the composition and structure of key communities in bacterial and fungal community structures of *S. miltiorrhiza* (Feng et al., 2018). This may be due to the difference in the type of fertilizer applied, which can notably influence microbial abundance and network complexity (Fan et al., 2019), thereby increasing the abundance of keystone species and radically changing the structure of the epiphytic microbial communities of *S. miltiorrhiza*.

In the Mantel correlation analysis of growth parameters and microbial community, no matter which site, the significance of microbial community and growth parameters in F3 was greater than that in other treatment groups, indicating that F3 treatment group had a more noteworthy effect on the epiphytic communities of *S. miltiorrhiza*. The medicinal components in the roots were markedly positively correlated with *Sphingobium*, *Mortierella*, *Fusarium*, and *Epicoccun*. The medicinal components of the stem were evidently positively correlated with *Novosphingobium* and *Dioszegia*, while the medicinal components of the leaves were clearly positively correlated with *Blautia* and *Genolevuria*. The variance decomposition showed that the hormones ABA, CTK, and GA were the main factors responsible for the changes in the epiphytic communities of *S. miltiorrhiza*. Studies have shown that *Sphingomonas* can improve plant resistance to drought, salt and alkali, and heavy metals, and can synthesize some plant hormones to promote plant growth (Haney et al., 2015). The *Fusarium* can produce plant-stimulating hormones (gibberellin), which increases crop yield. These keystone species possess the capability to promote plant nutrient uptake, and enhance plant tolerance to environmental stressors (Trivedi et al., 2020; Zhang et al., 2020).

In the Mantel correlation analysis showed that the influence of environmental factors on the rhizosphere bacteria of *S. miltiorrhiza* surpasses that on fungi, and these factors exert a stronger impact on the roots compared to other plant parts. Apparently, within the various treatment groups, the F3 treatment demonstrated a more pronounced influence on environmental factors in comparison to the other treatment groups. The phyla Proteobacteria, Actinobacteria, and Bacteroidetes showed a positive correlation with NO_3_^−^-N and a negative correlation with NH_4_^+^-N (Liu et al., 2023b). In addition to fertilization, these indicators are influenced by various other factors, such as soil pH (Wang et al., 2017), root exudate metabolites (Wu et al., 2017), and microbial diversity levels (Xun et al., 2019). Spatially different biotic and abiotic environmental factors may lead to different selection pressures between bacteria and fungi (Li et al., 2017b; Saleem et al., 2018). Keystone species significantly affected the process of community construction, likely because they exhibited high connectivity in community microbial networks and were good predictors of community deviation and turnover (Herren and McMahon, 2017, Herren and McMahon, 2018).

### 4.3 Relationship between niche epiphytic communities and quality of *S. miltiorrhiza*

Analysis of the epiphytic communities in niches showed that fertilization significantly affected some key species in the epiphytic communities of *S. miltiorrhiza*. In the bacterial co-occurrence network, Bacteroidota and Firmicutes were the dominant phyla, and other indices in the network increased, indicating that the bacterial community network was more stable after fertilization. In the fungal co-occurrence network, Basidiomycota, Mucoromycota, and Mortierellomycota were the dominant phyla, while other indices in the network decreased, showing that the fungal community network was no longer stable after fertilization. In the functional prediction, the protein functions of epiphytic bacterial communities were markedly enhanced. The function of pathogenic and saprophytic fungi was ​elevated, and that of commensal fungi was reduced. Mantel correlation analysis revealed heightened coefficients across all plant segments under the F3 treatment compared to other treatment cohorts, denoting F3 exerted a more pronounced impact on *S. miltiorrhiza* epiphytic populations. According to the variance decomposition, ABA, CTK, and GA were the main factors responsible for the changes in the epiphytic communities of *S. miltiorrhiza*. Studies have shown that *Sphingomonas* can improve the resistance of plants to drought, Salinization, heavy metals, and can synthesize some plant hormones to promote plant growth. *Fusariums* can produce plant stimulating hormone (GA), which can improve crop yield. These key species could enhance plant nutrient absorption, boost plant disease resistance, and improve plant stress tolerance (Trivedi et al., 2020; Zhang et al., 2020). Our work found that fertilization can select dominant and stable key species in the community, and then stimulate plants through hormones secreted by these key species to improve the yield and quality of *S. miltiorrhiza*. The effect of F3 treatment on the epiphytic communities was significant and gave the highest quality of *S. miltiorrhiza*. Therefore, we determined the best fertilization method is the combination of base fertilizer and foliar fertilizer.

## 5 Conclusion

In this study, we systematically examined the effects of fertility on community composition, species diversity, community assembly, and keystone species diversity of the epiphytic bacteria and fungi in *S. miltiorrhiza* from different ecological niches. The results showed that fertilization significantly affected the composition and abundance of the epiphytic microbial community of *S. miltiorrhiza*. The influence of compound fertilization on the bacterial community in various niches of *S. miltiorrhiza* was more remarkable than on the fungi. The microbial community construction of epiphytic bacteria and fungi in each part of the plant was dominated by deterministic processes. In the community, the *Sphingobium*, *Mortierella*, *Fusarium*, *Epicoccun*, and *Novosphingobium* exhibited an evident positive correlation with the medicinal components of *S. miltiorrhiza*. The hormones ABA, CTK, and GA were the main factors impacting the distribution difference of epiphytic colony in the *S. miltiorrhiza*. The combination of base fertilizer and foliar fertilizer was established as the optimal fertilization strategy.

## Data availability statement

The datasets presented in this study can be found in online repositories. The names of the repository/repositories and accession number(s) can be found in the article/**Supplementary Material**.

## Ethics statement

Ethics Committee approval was obtained from the Institutional Ethics Committee of Hebei University to the commencement of the study. The studies were conducted in accordance with the local legislation and institutional requirements. The participants provided their written informed consent to participate in this study.

## Author contributions

FG: Conceptualization, Data curation, Formal analysis, Funding acquisition, Investigation, Methodology, Project administration, Resources, Software, Supervision, Validation, Visualization, Writing – original draft, Writing – review & editing. CH: Data curation, Funding acquisition, Methodology, Supervision, Validation, Writing – original draft, Writing – review & editing. XL: Funding acquisition, Investigation, Methodology, Writing – review & editing. KW: Conceptualization, Formal analysis, Project administration, Resources, Software, Writing – original draft. ML: Conceptualization, Data curation, Formal analysis, Supervision, Writing – original draft. XZ: Data curation, Funding acquisition, Methodology, Writing – original draft. MX: Data curation, Formal analysis, Resources, Writing – original draft. XH: Data curation, Project administration, Software, Validation, Writing – original draft, Writing – review & editing.

## References

[B1] Almario J. Jeena G. Wunder J. Langen G. Zuccaro A. Coupland G.. (2017). Root-associated fungal microbiota of nonmycorrhizal Arabis alpina and its contribution to plant phosphorus nutrition. Proc. Natl. Acad. Sci. U.S.A. 114, E9403–E9412. doi: 10.1073/pnas.1710455114 28973917 PMC5676915

[B2] Banerjee S. Kirkby C. A. Schmutter D. Bissett A. Kirkegaard J. A. Richardson A. E. (2016). Network analysis reveals functional redundancy and keystone taxa amongst bacterial and fungal communities during organic matter decomposition in an arable soil. Soil Biol. Biochem. 97, 188–198. doi: 10.1016/j.soilbio.2016.03.017

[B3] Beckers B. Op De Beeck M. Weyens N. Boerjan W. Vangronsveld J. (2017). Structural variability and niche differentiation in the rhizosphere and endosphere bacterial microbiome of field-grown poplar trees. Microbiome. 5, 25. doi: 10.1186/s40168-017-0241-2 28231859 PMC5324219

[B4] Berendsen R. L. Pieterse C. M. Bakker P. A. (2012). The rhizosphere microbiome and plant health. Trends Plant Sci. 17, 478–486. doi: 10.1016/j.tplants.2012.04.001 22564542

[B5] Berg G. (2009). Plant-microbe interactions promoting plant growth and health: perspectives for controlled use of microorganisms in agriculture. Appl. Microbiol. Biotechnol. 84, 11–18. doi: 10.1007/s00253-009-2092-7 19568745

[B6] Bhattacharyya P. Roy K. S. Nayak A. K. Shahid M. Lal B. Gautam P.. (2017). Metagenomic assessment of methane production-oxidation and nitrogen metabolism of long term manured systems in lowland rice paddy. Sci. Total Environ. 586, 1245–1253. doi: 10.1016/j.scitotenv.2017.02.120 28238374

[B7] Bodenhausen N. Horton M. W. Bergelson J. (2013). Bacterial communities associated with the leaves and the roots of *Arabidopsis thaliana* . PLoS ONE. 8 (2), e56329. doi: 10.1371/journal.pone.0056329 23457551 PMC3574144

[B8] Bringel F. Couée I. (2015). Pivotal roles of phyllosphere microorganisms at the interface between plant functioning and atmospheric trace gas dynamics. Front. Microbiol. 6. doi: 10.3389/fmicb.2015.00486 PMC4440916 26052316

[B9] Buja L. M. Vander Heide R. S. (2016). Pathobiology of ischemic heart disease: past, present and future. Cardiovasc. Pathol. 25, 214–220. doi: 10.1016/j.carpath.2016.01.007 26897485

[B10] Chang Y. Chen F. Zhu Y. You Y. Cheng Y. Ma J. (2022). Influence of revegetation on soil microbial community and its assembly process in the open-pit mining area of the Loess Plateau, China. Front. Microbiol. 13. doi: 10.3389/fmicb.2022.992816 PMC9453671 36090080

[B11] Chen T. Nomura K. Wang X. Sohrabi R. Xu J. Yao L.. (2020). A plant genetic network for preventing dysbiosis in the phyllosphere. Nature. 580, 653–657. doi: 10.1038/s41586-020-2185-0 32350464 PMC7197412

[B12] Chen X. Q. Li T. Lu D. J. Cheng L. Zhou J. M. Wang H. Y. (2019). Estimation of soil available potassium in Chinese agricultural fields using a modified sodium tetraphenyl boron method. Land Degradation Dev. 31, 1737–1748. doi: 10.1002/ldr.3535

[B13] Cheng C. W. Chen L. Y. Chou C. W. Liang J. Y. (2015). Investigations of riboflavin photolysis via coloured light in the nitro blue tetrazolium assay for superoxide dismutase activity. J. Photochem. Photobiol. B. 148, 262–267. doi: 10.1016/j.jphotobiol.2015.04.028 25985146

[B14] Dai Z. Su W. Chen H. Barberán A. Zhao H. Yu M.. (2018). Long-term nitrogen fertilization decreases bacterial diversity and favors the growth of Actinobacteria and Proteobacteria in agro-ecosystems across the globe. Glob Chang Biol. 24, 3452–3461. doi: 10.1111/gcb.14163 29645398

[B15] Dangi S. Gao S. Duan Y. H. Wang D. (2020). Soil microbial community structure affected by biochar and fertilizer sources. Appl. Soil Ecol. 150, 103452. doi: 10.1016/j.apsoil.2019.103452

[B16] Dini-Andreote F. Stegen J. C. van Elsas J. D. Salles J. F. (2015). Disentangling mechanisms that mediate the balance between stochastic and deterministic processes in microbial succession. Proc. Natl. Acad. Sci. U. S. A. 112, E1326–E1332. doi: 10.1073/pnas.1414261112 25733885 PMC4371938

[B17] Douglas G. M. Maffei V. J. Zaneveld J. Yurgel S. N. Brown J. R. Taylor C. M.. (2020). PICRUSt2: An improved and customizable approach for metagenome inference. Nat. Biotechnol. 38, 685–688. doi: 10.1038/s41587-020-0548-6 32483366 PMC7365738

[B18] Erlacher A. Cernava T. Cardinale M. Soh J. Sensen C. W. Grube M.. (2015). Rhizobiales as functional and endosymbiontic members in the lichen symbiosis of Lobaria pulmonaria L. Front. Microbiol. 6. doi: 10.3389/fmicb.2015.00053 PMC4322706 25713563

[B19] Fan K. Delgado-Baquerizo M. Guo X. Wang D. Wu Y. Zhu M.. (2019). Suppressed N fixation and diazotrophs after four decades of fertilization. Microbiome. 7, 143. doi: 10.1186/s40168-019-0757-8 31672173 PMC6824023

[B20] Faust K. Raes J. (2012). Microbial interactions: from networks to models. Nat. Rev. Microbiol. 10, 538–550. doi: 10.1038/nrmicro2832 22796884

[B21] Feng M. M. Adams J. M. Fan K. K. Shi Y. Sun R. B. Wang D. Z.. (2018). Long-term fertilization influences community assembly processes of soil diazotrophs. Soil Biol. Biochem. 126, 151–158. doi: 10.1016/j.soilbio.2018.08.021

[B22] Fierer N. Bradford M. A. Jackson R. B. (2007). Toward an ecological classification of soil bacteria. Ecology. 88, 1354–1364. doi: 10.1890/05-1839 17601128

[B23] Flieder M. Buongiorno J. Herbold C. W. Hausmann B. Rattei T. Lloyd K. G.. (2021). Novel taxa of Acidobacteriota implicated in seafloor sulfur cycling. ISME J. 15, 3159–3180. doi: 10.1038/s41396-021-00992-0 33981000 PMC8528874

[B24] Fuhrman J. A. (2009). Microbial community structure and its functional implications. Nature. 459, 193–199. doi: 10.1038/nature08058 19444205

[B25] Gazdag O. Takács T. Ködöböcz L. Krett G. Szili-Kovács T. (2018). Alphaproteobacteria communities depend more on soil types than land managements. Acta Agric. Scand. Sect. B. 69, 147–154. doi: 10.1080/09064710.2018.1520289

[B26] Geng Y. Cao G. Wang L. Wang S. (2019). Effects of equal chemical fertilizer substitutions with organic manure on yield, dry matter, and nitrogen uptake of spring maize and soil nitrogen distribution. PloS One 14, e0219512. doi: 10.1371/journal.pone.0219512 31287845 PMC6615609

[B27] Guan S. Y. (1986). Soil enzymes and their research methods (in Chinese). Agric. Press Beijing pp, 273–339.

[B28] Guan Z. Lin D. Chen D. Guo Y. Lu Y. Han Q.. (2022). Soil microbial communities response to different fertilization regimes in young Catalpa bungei plantation. Front. Microbiol. 13. doi: 10.3389/fmicb.2022.948875 PMC9473346 36118227

[B29] Guo Y. B. Li Y. Xue L. M. Severino R. P. Gao S. H. Niu J. Z.. (2014). *Salvia miltiorrhiza*: An ancient Chinese herbal medicine as a source for anti-osteoporotic drugs. J. Ethnopharmacol. 155, 1401–1416. doi: 10.1016/j.jep.2014.07.058 25109459

[B30] Guo Z. B. Wan S. X. Hua K. K. Yin Y. Chu H. Y. Wang D. Z.. (2020). Fertilization regime has a greater effect on soil microbial community structure than crop rotation and growth stage in an agroecosystem. Appl. Soil Ecol. 149, 103510–103523. doi: 10.1016/j.apsoil.2020.103510

[B31] Hacquard S. SChadt C. W. (2015). Towards a holistic understanding of the beneficial interactions across the Populus microbiome. New Phytol. 205, 1424–1430. doi: 10.1111/nph.13133 25422041

[B32] Han Z. Q. Xu P. S. Li Z. T. Lin H. Y. Zhu C. Wang J. Y.. (2022). Microbial diversity and the abundance of keystone species drive the response of soil multifunctionality to organic substitution and biochar amendment in a tea plantation. Glob. Change Biol. Bioenergy. 14, 481–495. doi: 10.1111/gcbb.12926

[B33] Haney C. H. Samuel B. S. Bush J. Ausubel F. M. (2015). Associations with rhizosphere bacteria can confer an adaptive advantage to plants. Nat. Plants. 1, 15051. doi: 10.1038/nplants.2015.51 27019743 PMC4806546

[B34] Hautier Y. Niklaus P. A. Hector A. (2009). Competition for light causes plant biodiversity loss after eutrophication. Science. 324, 636–638. doi: 10.1126/science.1169640 19407202

[B35] Hector A. Schmid B. Beierkuhnlein C. Caldeira M. C. Diemer M. Dimitrakopoulos P. G.. (1999). Plant diversity and productivity experiments in european grasslands. Science. 286, 1123–1127. doi: 10.1126/science.286.5442.1123 10550043

[B36] Heiri O. Lotter A. F. Lemcke G. (2001). Loss on ignition as a method for estimating organic and carbonate content in sediments, reproducibility and comparability of results. J. Paleolimnol. 25, 101–110. doi: 10.1023/A:1008119611481

[B37] Herren C. M. McMahon K. D. (2017). Small subsets of highly connected taxa predict composi-tional change in microbial communities. Environ. Microbiol. 20, 2207–2217. doi: 10.1101/159087 29708645

[B38] Herren C. M. McMahon K. D. (2018). Keystone taxa predict compositional change in microbial communities. Environ. Microbiol. 20, 2207–2217. doi: 10.1111/1462-2920.14257 29708645

[B39] Iqbal A. He L. Ali I. Ullah S. Khan A. Khan A.. (2020). Manure combined with chemical fertilizer increases rice productivity by improving soil health, post-anthesis biomass yield, and nitrogen metabolism. PloS One 15, e0238934. doi: 10.1371/journal.pone.0238934 33027309 PMC7540855

[B40] Ives A. R. Carpenter S. R. (2007). Stability and diversity of ecosystems. Science. 317, 58–62. doi: 10.1126/science.1133258 17615333

[B41] Jiao S. Lu Y. (2020). Abundant fungi adapt to broader environmental gradients than rare fungi in agricultural fields. Glob Chang Biol. 26, 4506–4520. doi: 10.1111/gcb.15130 32324306

[B42] Kandeler E. Gerber H. (1988). Short-term assay of soil urease activity using colorimetric determination of ammonium. Biol. Fertil. Soils 6, 68–72. doi: 10.1007/BF00257924

[B43] Kentelky E. Szekely-Varga Z. (2021). Impact of foliar fertilization on growth, flowering, and corms production of five *gladiolus* varieties. Plants (Basel). 10, 1963. doi: 10.3390/plants10091963 34579495 PMC8466131

[B44] Khan N. Ali S. Shahid M. A. Mustafa A. Sayyed R. Z. Curá J. A. (2021). Insights into the Interactions among Roots, Rhizosphere, and Rhizobacteria for Improving Plant Growth and Tolerance to Abiotic Stresses: A Review. Cells. 10, 1551. doi: 10.3390/cells10061551 34205352 PMC8234610

[B45] Kielkopf C. L. Bauer W. Urbatsch I. L. (2020). Bradford assay for determining protein concentration. Cold Spring Harb. Protoc. 2020, 102269. doi: 10.1101/pdb.prot102269 32238597

[B46] Ley R. E. Peterson D. A. Gordon J. I. (2006). Ecological and evolutionary forces shaping microbial diversity in the human intestine. Cell. 124, 837–848. doi: 10.1016/j.cell.2006.02.017 16497592

[B47] Li F. Chen L. Zhang J. Yin J. Huang S. (2017a). Bacterial community structure after long-term organic and inorganic fertilization reveals important associations between soil nutrients and specific taxa involved in nutrient transformations. Front. Microbiol. 8. doi: 10.3389/fmicb.2017.00187 PMC5298992 28232824

[B48] Li W. T. Liu M. Jiang C. Y. Wu M. Chen X. F. Ma X. Y.. (2017b). Changes in soil aggregate-associated enzyme activities and nutrients under long-term chemical fertilizer applications in a phosphorus-limited paddy soil. Soil Use Manage. 33, 25–33. doi: 10.1111/sum.12322

[B49] Lin Y. Ye G. Kuzyakov Y. Liu D. Y. Fan J. B. Ding W. (2019). Long-term manure application increases soil organic matter and aggregation, and alters microbial community structure and keystone taxa. Soil Biol. Biochem. 134, 187–196. doi: 10.1016/j.soilbio.2019.03.030

[B50] Liu H. Awasthi M. K. Zhang Z. Syed A. Bahkali A. H. Sindhu R.. (2023a). Microbial dynamics and nitrogen retention during sheep manure composting employing peach shell biochar. Bioresour. Technol. 386, 129555. doi: 10.1016/j.biortech.2023.129555 37499921

[B51] Liu W. Cui S. Wu L. Qi W. Chen J. Ye Z.. (2023b). Effects of bio-organic fertilizer on soil fertility, yield, and quality of tea. J. Soil Sci. Plant Nutr. 23, 5109–5121. doi: 10.1007/s42729-023-01195-6

[B52] Lozupone C. A. Stombaugh J. I. Gordon J. I. Jansson J. K. Knight R. (2012). Diversity, stability and resilience of the human gut microbiota. Nature. 489, 220–230. doi: 10.1038/nature11550 22972295 PMC3577372

[B53] Lu T. Ke M. Lavoie M. Jin Y. Fan X. Zhang Z.. (2018). Rhizosphere microorganisms can influence the timing of plant flowering. Microbiome. 6, 231. doi: 10.1186/s40168-018-0615-0 30587246 PMC6307273

[B54] May R. M. (1998). How many species are there on Earth? Science. 241, 1441–1449. doi: 10.1126/science.241.4872.1441 17790039

[B55] McCann K. Hastings A. Huxel G. R. (1998). Weak trophic interactions and the balance of nature. Nature 395, 794–798. doi: 10.1038/27427

[B56] Mougi A. Kondoh M. (2012). Diversity of interaction types and ecological community stability. Science. 337, 349–3451. doi: 10.1126/science.1220529 22822151

[B57] Nguyen N. H. Song Z. Bates S. T. Branco S. Tedersoo L. Menke J.. (2016). FUNGuild: An open annotation tool for parsing fungal community datasets by ecological guild. Fungal Ecol. 20, 241–248. doi: 10.1016/j.funeco.2015.06.006

[B58] Niu J. H. Liu C. Huang M. L. Liu K. Z. Yan D. Y. (2021). Effects of foliar fertilization: a review of current status and future perspectives. J. Soil Sci. Plant Nutr. 21, 104–118. doi: 10.1007/s42729-020-00346-3

[B59] Olsen S. R. Cole C. V. Watanabe F. S. (1954). Estimation of Available Phosphorus in Soils by Extraction with Sodium Bicarbonate (Washington: Circular/United States Department of Agriculture).

[B60] Pande S. Merker H. Bohl K. Reichelt M. Schuster S. de Figueiredo L. F.. (2014). Fitness and stability of obligate cross-feeding interactions that emerge upon gene loss in bacteria. ISME J. 8, 953–962. doi: 10.1038/ismej.2013.211 24285359 PMC3996690

[B61] Pang H. Wu L. Tang Y. Zhou G. Qu C. Duan J. A. (2016). Chemical Analysis of the Herbal Medicine *Salviae miltiorrhizae* Radix et Rhizoma (Danshen). Molecules. 21, 51. doi: 10.3390/molecules21010051 26742026 PMC6273254

[B62] Ptacnik R. Solimini A. G. Andersen T. Tamminen T. Brettum P. Lepistö L.. (2008). Diversity predicts stability and resource use efficiency in natural phytoplankton communities. Proc. Natl. Acad. Sci. U. S. A. 105, 5134–5138. doi: 10.1073/pnas.0708328105 18375765 PMC2278227

[B63] Pu C. Yang G. Li P. Ge Y. Garran T. A. Zhou X.. (2022). Arbuscular mycorrhiza alters the nutritional requirements in *Salvia miltiorrhiza* and low nitrogen enhances the mycorrhizal efficiency. Sci. Rep. 12, 19633. doi: 10.1038/s41598-022-17121-2 36385104 PMC9668911

[B64] Ren J. Liu X. Yang W. Yang X. Li W. Xia Q.. (2021). Rhizosphere soil properties, microbial community, and enzyme activities: Short-term responses to partial substitution of chemical fertilizer with organic manure. J. Environ. Manage. 299, 113650. doi: 10.1016/j.jenvman.2021.113650 34481370

[B65] Sabir M. S. Shahzadi F. Ali F. Shakeela Q. Niaz Z. Ahmed S. (2021). Comparative effect of fertilization practices on soil microbial diversity and activity: an overview. Curr. Microbiol. 78, 3644–3655. doi: 10.1007/s00284-021-02634-2 34480627

[B66] Saleem M. Law A. D. Sahib M. R. Pervaiz Z. H. Zhang Q. M. (2018). Impact of root system ar-chitecture on rhizosphere and root microbiome. Rhizosphere 6, 47–51. doi: 10.1016/j.rhisph.2018.02.003

[B67] Schmidt S. K. Nemergut D. R. Darcy J. L. Lynch R. (2014). Do bacterial and fungal communities assemble differently during primary succession? Mol. Ecol. 23, 254–258. doi: 10.1111/mec.12589 26010467

[B68] Shi X. R. Ren B. B. Jiang L. L. Fan S. X. Cao Y. L. Ma D. R. (2021). Effects of organic manure partial substitution for chemical fertilizer on the photosynthetic rate, nitrogen use efficiency and yield of rice. Ying Yong Sheng Tai Xue Bao. 32, 154–162. doi: 10.13287/j.1001-9332.202101.021 33477223

[B69] Tamaki H. Wright C. L. Li X. Lin Q. Hwang C. Wang S.. (2011). Analysis of 16S rRNA amplicon sequencing options on the Roche/454 next-generation titanium sequencing platform. PloS One 6, e25263. doi: 10.1371/journal.pone.0025263 21966473 PMC3179495

[B70] Tarafdar J. Marschner H. (1994). Phosphatase activity in the rhizosphere and hyphosphere of VA mycorrhizal wheat supplied with inorganic and organic phosphorus. Soil Biol. Biochem. 26, 387–395. doi: 10.1016/0038-0717(94)90288-7

[B71] Tepper C. S. Gaynor S. C. (2015). Ribosomal internal transcribed spacer (ITS) DNA variation in millepora. J. Mar. Science: Res. Dev. 06, 1–6. doi: 10.4172/2155-9910

[B72] Ti C. Luo Y. Yan X. (2015). Characteristics of nitrogen balance in open-air and greenhouse vegetable cropping systems of China. Environ. Sci. pollut. Res. Int. 22, 18508–18518. doi: 10.1007/s11356-015-5277-x 26336846

[B73] Trivedi P. Leach J. E. Tringe S. G. Sa T. Singh B. K. (2020). Plant-microbiome interactions: from community assembly to plant health. Nat. Rev. Microbiol. 18, 607–621. doi: 10.1038/s41579-020-0412-1 32788714

[B74] Vandenkoornhuyse P. Quaiser A. Duhamel M. Le-Van A. Dufresne A. (2015). The importance of the microbiome of the plant holobiont. New Phytol. 206, 1196–1206. doi: 10.1111/nph.13312 25655016

[B75] Vorholt J. A. (2012). Microbial life in the phyllosphere. Nat. Rev. Microbiol. 10, 828–840. doi: 10.1038/nrmicro2910 23154261

[B76] Wang F. Ge S. Lyu M. Liu J. Li M. Jiang Y.. (2022b). DMPP reduces nitrogen fertilizer application rate, improves fruit quality, and reduces environmental cost of intensive apple production in China. Sci. Total Environ. 802, 149813. doi: 10.1016/j.scitotenv.2021.149813 34461469

[B77] Wang Y. Li C. Kou Y. Wang J. J. Tu B. Li H.. (2017). Soil pH is a major driver of soil diazotrophic community assembly in Qinghai-Tibet alpine meadows. Soil Biol. Biochem. 115, 547–555. doi: 10.1016/j.soilbio.2017.09.024

[B78] Wang J. L. Liu K. L. Zhao X. Q. Gao G. F. Wu Y. H. Shen R. F. (2022a). Microbial keystone taxa drive crop productivity through shifting aboveground-belowground mineral element flows. Sci. Total Environ. 811, 152342. doi: 10.1016/j.scitotenv.2021.152342 34919922

[B79] Wang M. Xu Y. Ni H. Ren S. Li N. Wu Y.. (2023). Effect of fertilization combination on cucumber quality and soil microbial community. Front. Microbiol. 14. doi: 10.3389/fmicb.2023.1122278 PMC9996052 36910239

[B80] Wardle D. A. (2006). The influence of biotic interactions on soil biodiversity. Ecol. Lett. 9, 870–886. doi: 10.1111/j.1461-0248.2006.00931.x 16796577

[B81] Wei X. Bai X. Cao P. Wang G. Han J. Zhang Z. (2023). Bacillus and microalgae biofertilizers improved quality and biomass of *Salvia miltiorrhiza* by altering microbial communities. Chin. Herb Med. 15, 45–56. doi: 10.1016/j.chmed.2022.01.008 36875436 PMC9975621

[B82] Wu X. Peng J. Liu P. Bei Q. Rensing C. Li Y.. (2021). Metagenomic insights into nitrogen and phosphorus cycling at the soil aggregate scale driven by organic material amendments. Sci. Total Environ. 785, 147329. doi: 10.1016/j.scitotenv.2021.147329 33940418

[B83] Wu H. Wang X. He X. Zhang S. Liang R. Shen J. (2017). Effects of root exudates on denitrifier gene abundance, community structure and activity in a micro-polluted constructed wetland. Sci. Total Environ. 598, 697–703. doi: 10.1016/j.scitotenv.2017.04.150 28456121

[B84] Xie L. He X. Wang K. Hou L. Sun Q. (2017). Spatial dynamics of dark septate endophytes in the roots and rhizospheres of Hedysarums coparium in northwest China and the influence of edaphic variables. Fungal Ecol. 26, 135–143. doi: 10.1016/j.funeco.2017.01.007

[B85] Xie F. Zhang B. C. Dai S. Jin B. B. Zhang T. Dong F. X. (2021). Efficacy and safety of *Salvia miltiorrhiza* (*Salvia miltiorrhiza* Bunge) and ligustrazine injection in the adjuvant treatment of early-stage diabetic kidney disease: A systematic review and meta-analysis. J. Ethnopharmacol. 281, 114346. doi: 10.1016/j.jep.2021.114346 34153447

[B86] Xiong C. He J. Z. Singh B. K. Zhu Y. G. Wang J. T. Li P. P.. (2021). Rare taxa maintain the stability of crop mycobiomes and ecosystem functions. Environ. Microbiol. 23, 1907–1924. doi: 10.1111/1462-2920.15262 32996254

[B87] Xun W. Li W. Xiong W. Ren Y. Liu Y. Miao Y.. (2019). Diversity-triggered deterministic bacterial assembly constrains community functions. Nat. Commun. 10, 3833. doi: 10.1038/s41467-019-11787-5 31444343 PMC6707308

[B88] Zhang J. Ai Z. Liu H. Tang D. W. S. Yang X. M. Wang G. L.. (2022). Short-term N addition in a Pinus tabuliformis plantation: microbial community composition and interactions show different linkages with ecological stoichiometry. Appl. Soil Ecol. 174, 104422. doi: 10.1016/j.apsoil.2022.104422

[B89] Zhang D. F. Cui X. W. Zhao Z. Zhang A. H. Huang J. K. Li W. J. (2020). Sphingomonas hominis sp. nov., isolated from hair of a 21-year-old girl. Antonie Van Leeuwenhoek. 113, 1523–1530. doi: 10.1007/s10482-020-01460-z 32783129

[B90] Zhao M. G. Chen L. Zhang L. L. Zhang W. H. (2009). Nitric reductase-dependent nitric oxide production is involved in cold acclimation and freezing tolerance in Arabidopsis. Plant Physiol. 151, 755–767. doi: 10.1104/pp.109.140996 19710235 PMC2754647

