# Supplementary material for: Impacts of fertilization methods on Salvia miltiorrhiza quality and characteristics of the epiphytic microbial community
Source: Front Plant Sci. 2024 May 16;15:1395628. doi: 10.3389/fpls.2024.1395628 (PMC11138495; doi:10.3389/fpls.2024.1395628)
Supplement: Supplementary file 1 [file DataSheet_1.docx]

Supplementary Material

**Impacts of fertilization methods on *Salvia miltiorrhiza* quality and characteristics of the epiphytic microbial community**

Feng Gong ^a,b^, Chao He ^c*^, Xianen Li ^c^, Kehan Wang ^a,b^, Min Li ^a,b^, Xiangyun Zhou ^a,b^, Minghui Xu ^a,b^, Xueli He ^a,b*^

^a^ College of Life Sciences, Hebei University, Baoding 071002, China

^b^ Key Laboratory of Microbial Diversity Research and Application of Hebei Province, No. 180, Wusidong Rd., Baoding 071002, China

^c^ Institute of Medicinal Plant Development, Chinese Academy of Medical Sciences & Peking Union Medical College, Beijing 100193, China

Author e-mail address: gf981120@126.com

^*^ Corresponding author: Chao He, Tel: +18801461215, E-mail address: hc891215@126.com; Xueli He, Tel: +13582075943, E-mail address: [xlh3615@126.com](mailto:xlh3615@126.com).

# Supplementary Figures and Tables

## Supplementary Tables

**Table S1** Data table of relative abundance of species in different parts of the plant

| **Classification level** | **Percentage** | | | |
| --- | --- | --- | --- | --- |
| **Bacteria，Phylum** | Proteobacteria | Bacteroidota | Firmicutes | Actinobacteriota |
| Y | 0.574384 | 0.135596 | 0.113063 | 0.132542 |
| J | 0.62982 | 0.161488 | 0.077849 | 0.084263 |
| G | 0.757781 | 0.045371 | 0.102281 | 0.061823 |
| **Bacteria，Genus** | *Pseudomonas* | *Hymenobacter* | *Sphingomonas* | *Rhizobium* |
| Y | 0.191444 | 0.082112 | 0.078049 | 0.005112 |
| J | 0.138252 | 0.092694 | 0.088817 | 0.013265 |
| G | 0.13537 | 0.008312 | 0.08976 | 0.075366 |
| **Fungi，Phylum** | Ascomycota | Basidiomycota | Mortierellomycota | Mucoromycota |
| Y | 0.730523 | 0.111545 | 0.001213 | 0.000083 |
| J | 0.68947 | 0.112142 | 0.001769 | 0.000137 |
| G | 0.490074 | 0.029482 | 0.012631 | 0.003771 |
| **Fungi，Genus** | *Alternaria* | *Cladosporium* | *Epicoccum* | *Dioszegia* |
| Y | 0.349824 | 0.207856 | 0.052502 | 0.028841 |
| J | 0.322163 | 0.179727 | 0.01956 | 0.021824 |
| G | 0.227576 | 0.102337 | 0.017947 | 0.002861 |

**Note:** Y, leaf. J, stem. G, root.

**Table S2** Plant and soil microbial α-diversity index values

| **Bacteria** | **chao1** | **shannon** | **goods_coverage** | **pielou_e** | **Fungi** | **chao1** | **shannon** | **goods_coverage** | **pielou_e** |
| --- | --- | --- | --- | --- | --- | --- | --- | --- | --- |
| GCK.1 | 873.878 | 7.6135337 | 0.975143403 | 0.8289721 | GCK.1 | 135 | 2.90969816 | 0.999930694 | 0.41301483 |
| GCK.2 | 863.8857 | 6.5985693 | 0.974506055 | 0.7323363 | GCK.2 | 168.5 | 3.4332311 | 0.999815183 | 0.47358246 |
| GCK.3 | 845.0938 | 7.9603665 | 0.978330147 | 0.8674529 | GCK.3 | 177.5 | 3.18983805 | 0.999861387 | 0.43040283 |
| GF1.1 | 689.913 | 7.4134188 | 0.984066284 | 0.8335223 | GF1.1 | 149 | 3.23027015 | 0.999838285 | 0.44924232 |
| GF1.2 | 379 | 5.359603 | 0.992192479 | 0.6529817 | GF1.2 | 182 | 3.38653857 | 0.999745876 | 0.45658094 |
| GF1.3 | 556.3704 | 6.415462 | 0.986615679 | 0.7365019 | GF1.3 | 165 | 3.19040951 | 0.999838285 | 0.44505469 |
| GF2.1 | 574.2609 | 6.4238705 | 0.984703633 | 0.751033 | GF2.1 | 163 | 3.7083702 | 0.999838285 | 0.50889891 |
| GF2.2 | 403.7143 | 7.1709879 | 0.994104525 | 0.8379726 | GF2.2 | 149.25 | 3.3938553 | 0.999930694 | 0.47009036 |
| GF2.3 | 395.6774 | 6.5863579 | 0.990121096 | 0.781251 | GF2.3 | 107 | 2.74078241 | 0.999884489 | 0.41910484 |
| GF3.1 | 802.7143 | 6.8195966 | 0.981038878 | 0.7706321 | GF3.1 | 194 | 3.95836919 | 0.999815183 | 0.54831056 |
| GF3.2 | 517.7143 | 6.4131807 | 0.988368388 | 0.7511624 | GF3.2 | 211 | 3.8335484 | 0.999815183 | 0.51455925 |
| GF3.3 | 787.1154 | 8.0325472 | 0.984384959 | 0.8723145 | GF3.3 | 208.75 | 3.16199306 | 0.999884489 | 0.41178253 |
| JCK.1 | 725 | 6.4380952 | 0.976099426 | 0.733703 | JCK.1 | 189 | 3.45489554 | 0.999699672 | 0.4640542 |
| JCK.2 | 850.3333 | 6.3453788 | 0.979764181 | 0.7263717 | JCK.2 | 177.25 | 3.20096369 | 0.999861387 | 0.43101979 |
| JCK.3 | 946.0233 | 7.5084016 | 0.974028043 | 0.812536 | JCK.3 | 208.5 | 3.66986207 | 0.999815183 | 0.47961022 |
| JF1.1 | 594.5 | 7.5941435 | 0.98820905 | 0.8498788 | JF1.1 | 202.6667 | 3.67866366 | 0.999630365 | 0.48550237 |
| JF1.2 | 531.5652 | 6.9222448 | 0.987571702 | 0.8002813 | JF1.2 | 208 | 4.30951443 | 0.999815183 | 0.56683025 |
| JF1.3 | 381.5 | 5.7476627 | 0.992989165 | 0.6895078 | JF1.3 | 172 | 3.41799789 | 0.999815183 | 0.46518635 |
| JF2.1 | 686.1875 | 6.5898181 | 0.984384959 | 0.7678887 | JF2.1 | 220 | 3.8394282 | 0.999745876 | 0.4972723 |
| JF2.2 | 757.8947 | 6.1260501 | 0.980242192 | 0.724742 | JF2.2 | 192.9091 | 3.0431931 | 0.999792081 | 0.40151964 |
| JF2.3 | 386.6875 | 6.8237998 | 0.993148502 | 0.8191276 | JF2.3 | 176 | 3.6601929 | 0.999815183 | 0.49218654 |
| JF3.1 | 524.2857 | 7.4202082 | 0.989165073 | 0.8422534 | JF3.1 | 231.875 | 3.93481244 | 0.999792081 | 0.50381067 |
| JF3.2 | 561.9655 | 3.459176 | 0.980560867 | 0.4202909 | JF3.2 | 310 | 4.26970832 | 0.999699672 | 0.52696596 |
| JF3.3 | 677.4839 | 6.4943528 | 0.979126832 | 0.7486114 | JF3.3 | 339 | 4.58222554 | 0.999792081 | 0.54760859 |
| YCK.1 | 1087 | 3.9887765 | 0.967176546 | 0.4493949 | YCK.1 | 190.5 | 3.45751179 | 0.999861387 | 0.45817693 |
| YCK.2 | 264 | 6.7902052 | 0.999681326 | 0.8457374 | YCK.2 | 138.5 | 3.26334246 | 0.999838285 | 0.4610196 |
| YCK.3 | 1390.038 | 8.8711196 | 0.965901848 | 0.9003187 | YCK.3 | 182 | 4.04400671 | 0.999768978 | 0.54110252 |
| YF1.1 | 1341.138 | 8.2062713 | 0.959528362 | 0.8453182 | YF1.1 | 228 | 3.9744178 | 0.999792081 | 0.51253643 |
| YF1.2 | 474.3333 | 5.9908443 | 0.988049713 | 0.7111876 | YF1.2 | 223 | 4.13163895 | 0.99967657 | 0.53456671 |
| YF1.3 | 1031.146 | 6.5771197 | 0.966698534 | 0.7110164 | YF1.3 | 174.5 | 3.23232691 | 0.999861387 | 0.43543485 |
| YF2.1 | 830.5 | 4.2437043 | 0.971797323 | 0.4790915 | YF2.1 | 189 | 3.06713696 | 0.99967657 | 0.41732015 |
| YF2.2 | 821.12 | 6.1872272 | 0.977852135 | 0.7034635 | YF2.2 | 220.5 | 3.52809618 | 0.999768978 | 0.45998185 |
| YF2.3 | 1076.053 | 6.8324256 | 0.977374124 | 0.7665548 | YF2.3 | 180.375 | 3.43788418 | 0.999792081 | 0.46455794 |
| YF3.1 | 570.1538 | 6.4834541 | 0.987253027 | 0.7610631 | YF3.1 | 266.1429 | 3.69102177 | 0.999653468 | 0.46367152 |
| YF3.2 | 594.12 | 6.2066897 | 0.984066284 | 0.7290709 | YF3.2 | 285 | 4.00161652 | 0.999537957 | 0.49746872 |
| YF3.3 | 479 | 6.829841 | 0.989802422 | 0.7971634 | YF3.3 | 257.3333 | 3.65708799 | 0.999607263 | 0.46044054 |

**Note:** Y, leaf. J, stem. G, root.

**Table S3** ANOSIM test for the significance of differences in bacterial and fungal community structure in different parts of roots and leaves of *Salvia miltiorrhiza*

| Comparison grouping | viruses | | fungi | |
| --- | --- | --- | --- | --- |
|  | R | P | R | P |
| YCK-YF1 | 0.3333 | 0.9353 | 0.1851 | 0.2885 |
| YCK-YF2 | 0.6666 | 0.1592 | 0.074 | 0.4776 |
| YCK-YF3 | 0.5555 | 0.0895 | 0.7777 | 0.0845 |
| YF1-YF2 | 0.5925 | 0.0845 | 0.1481 | 0.398 |
| YF1-YF3 | 0.6666 | 0.1194 | 0.6296 | 0.0945 |
| YF2-YF3 | 0 | 0.5174 | 0.7777 | 0.0746 |
| JCK-JF1 | 0.2222 | 0.3184 | 0.074 | 0.4626 |
| JCK-JF2 | 0.8888 | 0.1393 | -0.074 | 0.8308 |
| JCK-JF3 | 0.9259 | 0.1144 | 0.6666 | 0.0597 |
| JF1-JF2 | 1 | 0.1094 | 0.5925 | 0.1194 |
| JF1-JF3 | 0.9259 | 0.0945 | 0.7777 | 0.0995 |
| JF2-JF3 | 0 | 0.4776 | 0.6296 | 0.1194 |
| GCK-GF1 | 0.6296 | 0.0845 | 0.037 | 0.3532 |
| GCK-GF2 | 1 | 0.1144 | -0.1481 | 0.8855 |
| GCK-GF3 | 0.9259 | 0.0995 | 0.037 | 0.4925 |
| GF1-GF2 | 0.5925 | 0.0845 | 0.1851 | 0.2437 |
| GF1-GF3 | 0.8148 | 0.0995 | 0.1481 | 0.3681 |
| GF2-GF3 | 0.6296 | 0.1194 | 0.2222 | 0.2985 |

**Note:** Y, leaf. J, stem. G, root.

## Supplementary Figures

**Fig. S1** Dilution curves of Danshen epibiotic bacteria (a) and fungi (b) OTUs. Y, leaf. J, stem. G, root.


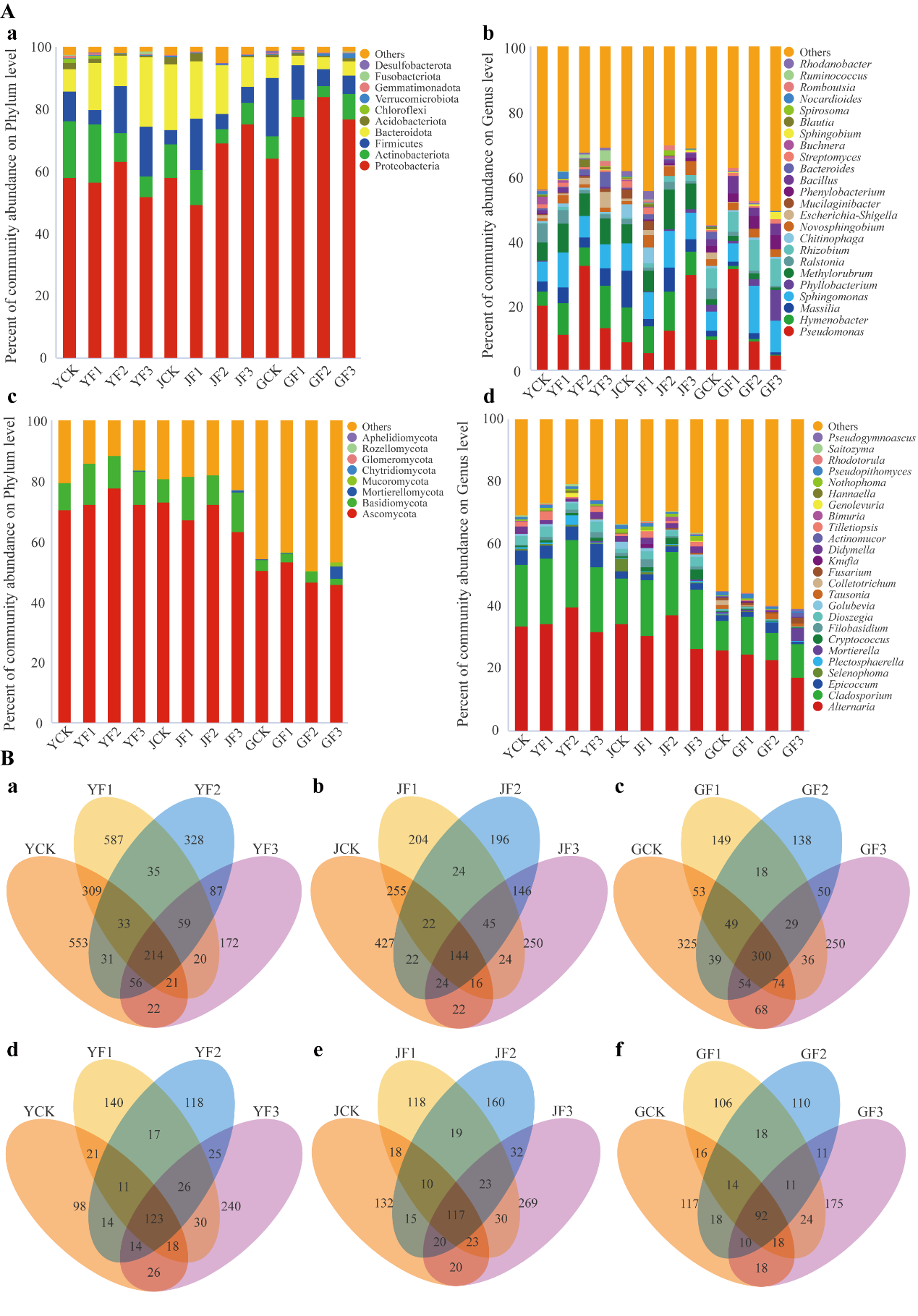
 **Fig. S2** Relative abundance map and VENN plot of *Salvia miltiorrhiza* epibiotic species at phylum and genus taxonomic levels. A, a, level of phylum bacteria. b, level of genus bacteria. c, level of phylum fungi. d, level of genus fungi. B, Leaf (a) stem (b) and root (c) bacteria; leaf (d) stem (e) and root (f) fungi. Y, leaf. J, stem. G, root.


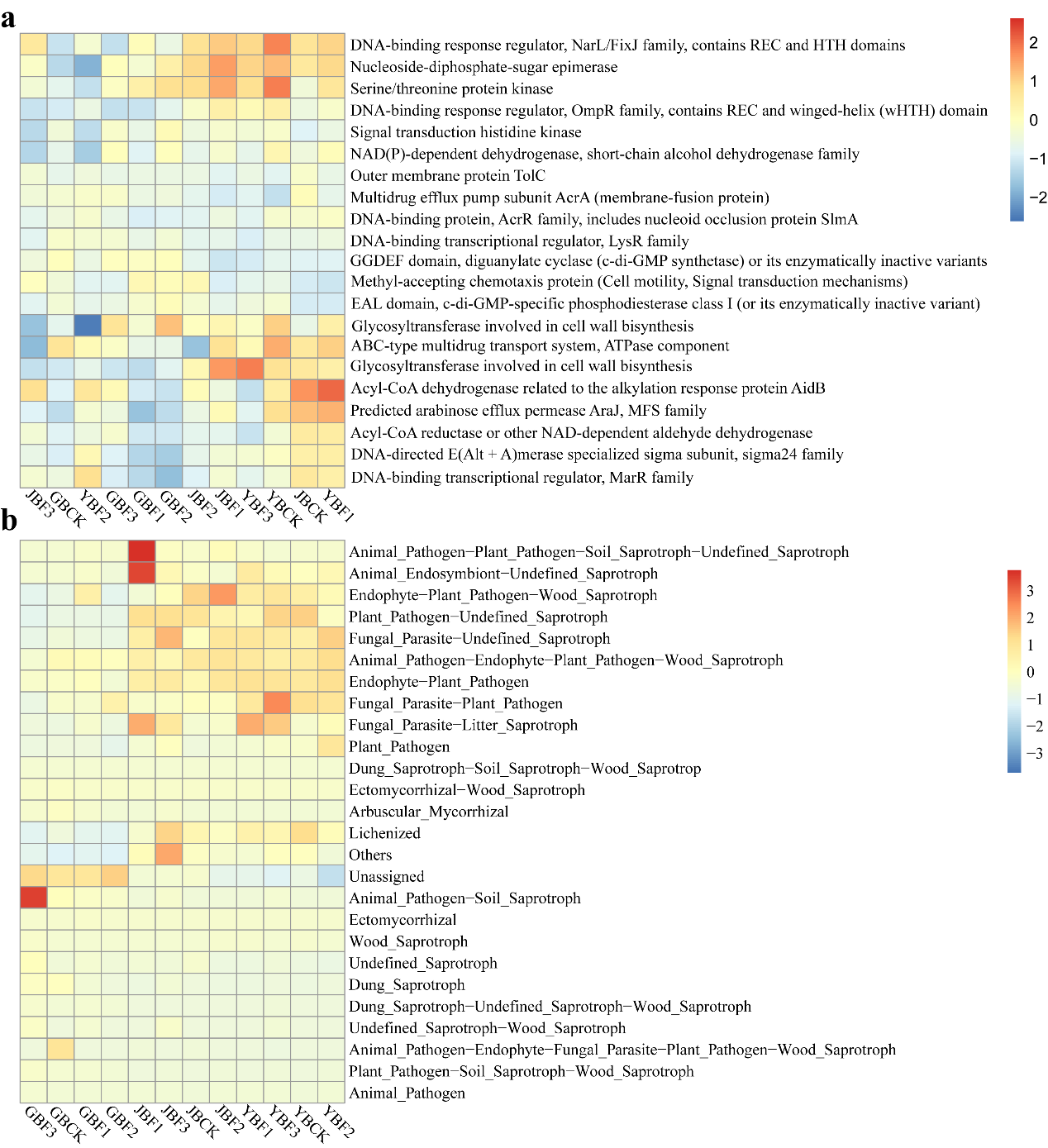
 **Fig. S3** Heat map of functional predicted abundance clustering of epibiotic bacteria (a) and fungi (b) in *Salvia miltiorrhiza* under different fertilization treatments. Y, leaf. J, stem. G, root. Y, leaf. J, stem. G, root.
